# Low Vision Rehabilitation and Eye Exercises: A Comprehensive Guide to Tertiary Prevention of Diabetic Retinopathy

**DOI:** 10.3390/life15060857

**Published:** 2025-05-26

**Authors:** Tibor Rák, Andrea Kovács-Valasek, Etelka Pöstyéni, Róbert Gábriel, Adrienne Csutak

**Affiliations:** 1 Department of Ophthalmology, Medical School, University of Pécs, Szigeti út 12, 7624 Pécs, Hungary; rak.tibor@pte.hu (T.R.); csutak.adrienne@pte.hu (A.C.); 2 Department of Pharmacognosy, Faculty of Pharmacy, University of Pécs, Rókus utca 2, 7624 Pécs, Hungary; 3 Department of Neurobiology, Faculty of Sciences, University of Pécs, Ifjúság útja 6, 7624 Pécs, Hungary; valasek@gamma.ttk.pte.hu (A.K.-V.); etelka14@gmail.com (E.P.); 4 János Szentágothai Research Centre, University of Pécs, Ifjúság útja 20, 7624 Pécs, Hungary

**Keywords:** diabetic retinopathy, low vision rehabilitation, visual training, Bates–Schneider method, animal-assisted therapy, ocular yoga, orthoptic exercises, physical exercise

## Abstract

Diabetic retinopathy (DR) is a leading cause of vision loss in patients with diabetes. While medical treatments like retinal laser photocoagulation, anti-VEGF therapy, and vitrectomy are primary, complementary therapies are gaining increasing attention. Based on the existing literature, a healthy lifestyle, including a balanced diet, stress management techniques, and regular physical activity targeting DR, can help regulate blood sugar levels and improve overall physical and mental health to reduce complications. This article explores physical activities and visual training methods related to DR, emphasizing complementary therapies, even though some of these practices are currently not fully integrated into evidence-based ophthalmology. Low vision exercises and aids help patients make the most of their remaining vision, improving their ability to perform everyday tasks, reducing the impact of vision loss, and promoting independence. There is some evidence that eye-related physiotherapy can improve the quality of life for patients with DR, although selection bias cannot be excluded in the presented studies. Consistent physical activity promotes holistic health, and therapies should be regularly monitored by ophthalmologists. This review further helps integrative healthcare professionals in offering appropriate therapies for rehabilitation purposes in the treatment of ophthalmic diseases, particularly DR.

## 1. Introduction

Diabetic retinopathy (DR) is a leading cause of vision loss in people with diabetes, affecting millions worldwide [1,2]. According to precise estimates by the Vision Loss Expert Group of the Global Burden of Disease Study and the Global Burden of Disease 2019 Blindness and Vision Impairment Collaborators, approximately 1.07 million people were affected by blindness and nearly 3.28 million had moderate to severe visual impairment globally due to DR [3]. Proper management of diabetes, including blood sugar control, stress management, and physical activity, is crucial in preventing the progression of this condition [4,5]. This ocular condition is primarily managed through medical interventions such as retinal laser photocoagulation, anti-VEGF therapy, and vitrectomy [6]. A significant number of patients with diabetic retinopathy experience varying degrees of vision impairment, the management of which presents a considerable challenge to ophthalmology [7]. However, treatments such as anti-VEGF therapy and certain surgeries can improve vision in many cases. Recent studies have demonstrated the efficacy of anti-VEGF therapies and certain surgical interventions in improving vision in patients with severe visual impairment [7,8]. The Diabetic Retinopathy Clinical Research (DRCR) Network found that intravitreal anti-VEGF injections significantly improve vision in diabetic macular edema patients [8]. Furthermore, systematic reviews of cataract surgeries have shown substantial visual improvement, particularly in high-income countries [9,10]. Combined cataract surgery and visual rehabilitation have also been reported to enhance visual acuity and quality of life in visually impaired individuals [9].

However, complementary therapies like meditation, yoga, and mindfulness are gaining attention for their potential benefits in managing diabetes and its complications, including DR [6]. Evidence suggests that the adoption of a healthy lifestyle, including a balanced diet rich in antioxidants and regular physical activity, can significantly impact the progression of DR [5,6,11]. Furthermore, stress management techniques, such as yoga and meditation, can help control blood sugar levels and reduce the risk of complications [6]. According to the results of Amore et al. using data from the Italian Ministry of Health, 19% of low vision specialists were orthoptists [12]. Italian statistics also demonstrate a slightly higher involvement of orthoptists (2.6%) than ophthalmologists (1.2%) in low vision rehabilitation care [12]. This ratio may be comparable to that of other developed countries, indicating that visually impaired DR patients are likely to utilize visual training services more frequently, although precise statistics are unfortunately lacking in several areas. Fitzmaurice noted that in Australia, orthoptists are skilled in providing low vision rehabilitation training, and patients experience high levels of both subjective and objective satisfaction [13]. Raphanel et al. mentioned examples of actual low vision practice in other countries: despite the current recommendations outlined by the French High Health Authority, only 10–15% of French visually impaired patients are referred to a low vision professional, while it is estimated that 90% of patients could benefit from this service [14]. In India, only 30% of eligible patients are referred for low vision rehabilitation [14]. In the UK, there is often a mismatch between what treatments eye care professionals believe are available and what visually impaired patients actually receive [14]. In Canada, there is neither a standardized low vision rehabilitation model nor a consistent referral system across different regions [14]. Based on the above data, there is a significant global demand for accessible rehabilitation options for DR patients. Additionally, given the high workload of ophthalmologists, orthoptists and visual therapists can play a crucial role in the rehabilitation and mental well-being of patients. Collaboration between other interdisciplinary healthcare professionals is advisable. As a result of teamwork, lifestyle suggestions can be planned and maintained together with the involvement of family members and friends [15]. Integrating a multifaceted lifestyle medicine approach, which includes medical treatments, lifestyle changes, and the use of low vision aids and visual exercises, can significantly enhance the quality of life for patients by maximizing their remaining vision and promoting independence.

In the following sections, the best known physical activities and visual training practices and their relation to DR are explained. As the practice of eye-related exercises has become very popular, it is important that ophthalmologists, visual trainers (or orthoptists), opticians, and optometrists are familiar with complementary therapies. In the following sections, the authors elaborate on different methods and their relevance in ophthalmic diseases.

## 2. Methods

### 2.1. Search Strategy

A systematic literature review and meta-analysis were conducted to assess the efficacy and scope of complementary and rehabilitative interventions in the tertiary prevention of DR. The search was performed in four major databases for studies published between 1969 and 2025: PubMed, Scopus, Web of Science, and Google Scholar. The search strategy employed both MeSH terms and free-text keywords derived from the concepts covered in this review article. The final search string included combinations of the following keywords: (“diabetic retinopathy” OR “DR”) AND (“low vision rehabilitation” OR “visual training” OR “orthoptic exercises” OR “ocular physiotherapy” OR “ocular yoga” OR “animal-assisted therapy” OR “guide dog” OR “diabetic alert dog” OR “hippotherapy” OR “Bates method” OR “Schneider method” OR “capillary exercise” OR “Chinese eye exercise” OR “office exercises” OR “Trataka” OR “Kolpakov gymnastics” OR “Katsuzo Nishi” OR “Zalmanov principle” OR “eurhythmy”/“Eurhythmie” OR “mind-body therapy” OR “meditation” OR “Shinrin-Yoku”/“forest bathing” OR “psychosomatic”). In addition, a hand search of references in key review articles was performed to identify any studies not captured by the database searches.

### 2.2. Study Selection and Data Extraction

Two independent reviewers screened the titles and abstracts. After duplicate removal, the full texts of potentially relevant articles were assessed. Disagreements were resolved through discussion or consultation with a third expert.

Inclusion criteria were as follows:

▪ Clinical studies (RCTs, controlled before–after studies, and cohort studies) evaluating low vision rehabilitation or complementary mind–body therapies in patients with DR or diabetic visual impairment;
▪ Studies reporting visual, psychological, or metabolic outcomes;
▪ Interventions including eye-focused exercises, physical activity, animal-assisted rehabilitation, yoga, or integrated lifestyle medicine protocols.

Exclusion criteria were as follows:

▪ Studies focused exclusively on pharmacologic or surgical management (e.g., anti-VEGF and laser therapy);
▪ Non-clinical studies, editorials, conference abstracts, or letters;
▪ Populations with non-diabetic causes of visual impairment.

Data extraction included the following:

▪ Study design and country;
▪ Sample size and patient demographics;
▪ Type, duration, and frequency of intervention;
▪ Outcome measures, e.g., visual acuity (VA), quality of life (QoL), blood glucose metrics (HbA1c), contrast sensitivity, and psychosocial scales;
▪ Results and reported effect sizes.

When necessary, corresponding authors were contacted for missing data.

The methodological quality of included studies was assessed descriptively. Particular attention was paid to selection bias, especially in studies involving lifestyle interventions or non-blinded designs (e.g., yoga, dog-assisted therapy, and office exercises). Studies with a high risk of bias were excluded.

## 3. Evidence-Based Low Vision Rehabilitation in Diabetic Vision Loss

Visual impairment or low vision (caused by DR) is a type of vision loss that cannot be further corrected with standard glasses, contact lenses, medical treatment, or surgery, and it significantly and permanently affects an individual’s daily life in most cases [16]. Low vision rehabilitation methods (Table 1) are designed to help individuals with visual impairments make the most of their remaining vision as tertiary prevention. These exercises can improve visual function, enhance contrast sensitivity, and reduce strain on the eyes. For DR patients, low vision exercises can be particularly beneficial in maintaining visual acuity and adapting to changes in vision [17]. Contrast sensitivity training that involves distinguishing between different levels of contrast can help improve an individual’s ability to see in low-light conditions and recognize objects against varying backgrounds [17]. This is crucial for patients with DR, who often experience reduced contrast sensitivity. For patients with central vision loss, training the eccentric and peripheral vision can help improve overall visual awareness [17]. Exercises that encourage the use of peripheral vision can aid in navigating environments and performing daily tasks more effectively. Low vision aids are specialized devices designed to assist individuals with visual impairments in maximizing their remaining vision [16,17]. These aids can significantly improve the ability to perform daily activities, such as reading, writing, and recognizing faces, which are challenging for DR patients [16]. Handheld or stand magnifiers and telescopic devices can enlarge text and images, making it easier for patients to read and perform detailed tasks. Electronic magnifiers, which offer adjustable magnification and contrast settings, provide additional flexibility and convenience [16,17]. For example, a clip-on magnifier lens allows both hands of the patient to be free while self-dosing the prescribed insulin [17]. These are also particularly useful for activities such as watching television, attending events, or navigating outdoor environments. Electronic aids such as screen readers, text-to-speech software, and electronic magnifiers can assist patients in accessing digital content and performing computer-based tasks [17]. These aids can enhance independence and productivity in both personal and professional settings. Proper adaptive lighting is essential for maximizing visual function: adjustable lamps, task lighting, and devices that enhance contrast can help reduce eye strain and improve visibility in various environments [17]. The integration of low vision exercises and aids into management plans for DR patients can have a profound impact on their quality of life. These interventions not only help patients adapt to their visual impairments but also empower them to maintain independence and engage in daily activities.

### 3.1. Animal-Assisted Therapy in Blindness from Diabetic Retinopathy

According to Bassan et al., there are approximately 500,000 service dogs in the United States; however, only about 2% of blind or visually impaired individuals keep them, highlighting the need for increased access to and availability of these trained animals [21]. The border between animal-assisted therapy interventions and assistance animals is not consistently clear due to subjective and psychosocial factors. The former has therapeutic value, while the latter is a low vision rehabilitation method [22]. A scoping review highlighted that animal-assisted interventions can enhance well-being and function in hospital rehabilitation settings. These interventions, which often involve officially certified dogs, have shown improvements in social and emotional well-being, ambulation, motor skills, and verbal communication [20,22,23]. Dogs generally provide companionship and emotional support, which can significantly reduce feelings of isolation and depression often experienced by individuals with visual impairments. Lundquist et al. stated that dog-assisted interventions can have positive effects in healthcare settings, including for patients with cognitive and psychiatric conditions [24]. Guide dogs are trained to help navigate obstacles, enhancing the mobility and independence of their handlers [23]. The findings of Glenk et al. show that blind people, compared to non-dog owners, with a guide dog in Austria are more likely to believe that service animals provide more benefits regarding their health and psychosocial status [23]. A total of 93% of respondents reported that their guide dog’s importance was the same as that of their relatives, and they were generally satisfied with the dog’s work [23]. It is also known in the literature that visually impaired individuals, regardless of age, prefer to keep guide dogs despite the availability of the most modern digital navigation devices or mobility aids [23]. Although the literature on guide dogs does not specifically address connections with patients who are blind due to diabetes, diabetic alert dogs offer a more interesting solution for them [25]. Keeping these dogs can be beneficial for minors and adolescents with type 1 diabetes and older patients with type 2 diabetes as they not only read their owner’s physical body signals but also olfactorily detect hypoglycemia (0.29–0.80 g/dL) and hyperglycemia (0.49–0.96 g/dL) [25]. Lippi and Lebani concluded that the current published evidence may contribute to undeniable health and psychological benefits for diabetic people. However, their efficiency in detecting harmful, even life-threatening, blood glucose variations remain questionable [25]. Additionally, there is no data in the literature on diabetic retinopathy and diabetic alert dogs. Equine-assisted therapy (or hippotherapy) has shown promising benefits for individuals with blindness or visual impairments. This intervention can provide a multisensory experience that stimulates various senses, compensating for the lack of visual input. The rhythmic and repetitive movements of the horse can help improve balance, coordination, and core strength, which are often areas of concern for those with visual impairments [26]. Additionally, the bond formed between the patient and the horse can foster a sense of trust, confidence, and emotional stability. This connection is particularly beneficial for individuals who may experience anxiety or depression due to their visual impairment. Moreover, it can enhance spatial awareness and orientation skills as patients learn to navigate and interact with their environment in new ways. The therapeutic setting also offers a safe and supportive space for patients to explore their capabilities and build resilience [26]. The authors did not find any studies in the literature describing the real connection between hippotherapy and DR. However, Klimova et al. conducted a unique study with children aged 7–13 years with type 1 diabetes [27]. According to their results, during hippotherapy rehabilitation performed twice a week, the children’s blood glucose levels significantly normalized after an initial increase [27]. The psychological assessment also showed that children were emotionally bonded with the animals, which could make intervention beneficial. Based on their results, Klimova et al. found hippotherapy to be more advantageous for young diabetic patients compared to other rehabilitation methods [27]. Although canines and horses are commonly used for therapeutic purposes, several other animal species can also be utilized (e.g., feline-assisted therapy or ornithotherapy). These benefits can extend to ophthalmic patients, especially DR patients, by providing emotional support and improving their overall well-being. While there is growing interest in animal assisted therapy, some studies highlight the need for more rigorous research to fully understand its benefits and mechanisms on DR patients and integrating these therapies into standard care practices. Another limitation of this intervention is its high financial burden. The training, certification, and living costs for a guide dog range between EUR 34,000 and 40,000 [23], while the costs of a diabetic alert dog depend on their training organization, ranging between EUR 1500 and 18,500 [25].

### 3.2. Orthoptic Exercises in Diabetic Eyes

Orthoptic exercises were originally evaluated for the management of heterophoria, intermittent strabismus, convergence insufficiency, and presbyopic accommodative disorders [28], but the following causes give a rationale in diabetic patients too. Studies indicate that approximately 1–14% of diabetics experience ocular motor nerve palsies during the course of their disease [29]. This condition is significantly more common in diabetic individuals compared to non-diabetic individuals, with the incidence being 5–10 times higher [29]. Individuals with DR or diabetic nephropathy face a heightened risk of developing ophthalmoplegia [30]. While DR primarily affects the retina, the same vascular complications can also impact the nerves controlling the extraocular muscles, leading to conditions such as cranial mononeuropathy [30]. Isolated cranial nerve palsy is approximately seven times more prevalent among diabetic patients, likely due to microvascular infarction [30,31]. The third cranial nerve is most commonly affected [29,31,32], but the fourth and sixth cranial nerves can also be involved [29,31], with functional recovery typically occurring within six months [33]. Although multiple cranial nerve palsies are rare in diabetes, they necessitate neuroimaging to exclude compressive lesions [30]. Oculomotor nerve palsies in diabetic patients can lead to symptoms such as diplopia and ptosis [17,29]. Idiopathic eyelid ptosis (a disorder of the third cranial nerve) is linked to an increased risk of insulin resistance [34]. Sensory neuropathy of the fifth cranial nerve can cause corneal hypesthesia, heightening the risk of dry eye and neurotrophic keratitis [35]. Regular blinking exercises can help patients become more aware of their blinking, supplemented by artificial eye drops [36]. Raskind reports the main characteristics of diabetes-induced convergence insufficiency causing diplopia and other visual disturbances [37]. The results of Rundström and Eperjesi indicate the necessity of evaluating the binocular vision of low vision patients (e.g., DR patients) to detect orthoptic disorders [38]. Treatment of convergence insufficiency aims to improve convergence exercises by pencil push-ups (accommodative orthoptic exercise by using a pencil), near-distance fixation changes, stereograms, etc. [32]. According to Raskind, systemic convergence insufficiency should primarily be managed using prismatic and additive lenses [37]. Orthoptic exercises and strabismus surgery is indicated if the misalignment is stable and long-standing and if the patients do not tolerate prismatic glasses [32].

Macfarlene et al. reports that there are limited data on the convergence training of stroke patients in the literature. It is important to mention this neurovascular condition because they also emphasize that diabetic patients, especially with visual impairments such as DR or cataract, are generally more vulnerable to stroke [39]. According to their study, the outcomes support the benefits of orthoptic interventions; therefore, a visual trainer/orthoptist is an essential part in teamwork [39]. Maagard et al. reported that vergence exercises (e.g., Brock’s string fusion, picture fusion using an aperture ruler, etc.) induce faster recovery of convergence insufficiency than accommodation exercises (e.g., distance-to-near accommodative visual acuity cards, etc.) in school children [40]. Horwood and Toor found that separating convergence and accommodation exercises seemed more effective than training both functions concurrently [28]; therefore, it is wiser to choose one type of training method to improve the patient’s orthoptic skills. Yadav et al. compared the efficacy of pencil push-up vs. office-based orthoptic therapy on patients with asthenopic symptoms due to convergence insufficiency [41]. Unfortunately, they excluded diabetes-related convergence insufficiency from their study, although useful conclusions could have been drawn.

Orthoptic exercises could have limitations and complications, such as pain, subconjunctival suffusion, and suture rupture in postoperative cases [42]. Zeng et al. used postoperative rehabilitation exercises in a study on postoperative patients with blow-out orbital fracture with retrobulbar anesthesia [42], but this can be interpreted on pars plana vitrectomy on DR. Therefore, we discourage vitrectomy patients from performing orthoptic exercises postoperatively to avoid severe complications. Another limitation of these exercises is the clinically rare phenomenon, termed horror fusionis, derived from Bielschowsky, which refers to a specific clinical syndrome where patients’ extraocular muscles actively avoid bifoveal fixation despite all attempts to achieve stereoscopic vision. It is an acquired disorder of central fusion, which causes diplopia in all positions of gaze, leading the patient to experience anxiety because even with correction, both eyes could neither fuse nor suppress images. Orthoptic visual therapists should warn their patients not to perform any orthoptic exercises on their own without medical professional guidance and diagnosis [43].

## 4. Physical Activity—Evidence in Ophthalmology

DR is a common complication of diabetes, characterized by damage to the retinal blood vessels due to prolonged hyperglycemia [6]. Among these, whole-body exercises and physical activity play a crucial role in both the prevention and management of DR [6,44]. These interventions directly impact ocular health through several physiological mechanisms: Regular physical activity enhances cardiovascular health, leading to better blood flow and oxygen delivery to the retina. Improved circulation helps maintain the integrity of retinal blood vessels and reduces the risk of ischemic damage [44]. Regular exercise improves endothelial function and increases ocular perfusion, which can have direct benefits for eye health by enhancing blood flow to ocular tissues [45]. Physical activity increases insulin sensitivity and helps regulate blood glucose levels. Better glycemic control is essential for preventing the progression of DR as high blood sugar levels are a primary cause of retinal damage [5,6,44]. Exercise induces the production of antioxidant enzymes and reduces systemic inflammation. Lower levels of oxidative stress and inflammation protect retinal cells from damage and slow the progression of DR [6,46]. Regular physical activity improves mitochondrial function and energy production in retinal cells. Enhanced mitochondrial function supports cellular health and resilience against metabolic stress [44]. Over time, diabetes damages peripheral circulation, thus causing changes in the eyes, kidneys, extremities, brain, etc. Based on Zalmanov’s “peripheral disease” principle, it is important for diabetic patients to perform capillary exercise every day, as well as exercises that stimulate the circulation of the limbs and help preserve long-term visual functions. Whole-body vibration increases the expression of fibronectin type III domain-containing protein 5 and brain-derived neurotrophic factor (BDNF) transcriptional factors, which are neuroprotective and enhance muscle contraction and relaxation [6,47]. These factors play a role in maintaining retinal health and preventing neurodegeneration [5,6,44]. Maintaining a healthy weight and cardiovascular health through exercise reduces the risk of comorbid conditions, such as hypertension and hyperlipidemia, which can exacerbate DR [6]. The Centers for Disease Control and Prevention, the World Health Organization (WHO), and the American Heart Association (AHA) all recommend 150 min of moderate aerobic exercise per week, which is equivalent to about 30 min per day for five days per week. This can include walking, cycling, swimming, dancing, and even active gardening [48].

Whole-body exercises and physical activity are integral components of a comprehensive approach to managing DR. Incorporating regular exercise into management plans for individuals with diabetes can significantly reduce the risk and progression of DR, ultimately preserving vision and enhancing quality of life.

### 4.1. Office-Based Exercises and Diabetic Retinopathy

The modern sedentary lifestyle and workplace environment are posing new difficulties to people’s health, notably their eyes and visual function. Prolonged near work and screen time in the office put significant strain on the eyes, resulting in a variety of health complications [49,50]. Continuous near concentrating can lead to eye fatigue, dry eye syndrome, and visual impairment [49,50]. In the worst-case scenario, both extended hours of near labor and the resulting chronic dry eye have been linked to an increased risk of depression and, in severe cases, suicide [36,51]. These rates are similarly higher among individuals with DR compared to the general population [52,53]. A study from the Kangbuk Samsung Cohort found that adults with prediabetes who worked more than 52 h per week had a significantly higher risk (hazard ratio of 2.00) of developing type 2 diabetes compared to those working 35–40 h per week [54]. Research published in Diabetes Care indicates that individuals with diabetes are less likely to be employed and, if employed, tend to have more work-loss days and health-related work limitations. This study did not find a significant change in weekly hours worked due to diabetes [55]. Another study highlighted that certain work-related factors, such as long working hours and shift work, could increase the risk of cardiovascular diseases in workers with diabetes, which can be related to diabetic complications like retinopathy [56]. Recently, increasing regional and international recommendations for various office gymnastics have been developed with the primary aim of reducing eye and mental fatigue. Additionally, these exercises have the secondary goal of alleviating stress caused by prolonged screen work, contributing to overall physical and mental well-being [36,57], which is also essential to diabetic and DR patients. The exercises of the Bates–Schneider Method include palming (covering the eyes with the palms) and regular blinking, which are basic relaxation techniques [57,58]. Occupational health workers and the American Academy of Ophthalmology (AAO) recommend the 20/20/20 rule for computer workers [36,59,60], which involves taking a 20 s break to look at something 20 feet (6 m) away every 20 min, and the regular use of preservative-free artificial tears [36], although there are controversial results in its efficacy [60]. In Hungary and Austria, “12 screen Tibetans”, developed by Martin Donner, an orthopedic surgeon, was promoted in online recommendations and publications for occupational health to interrupt computer office work by performing simple gymnastics to counteract the lack of movement and prevent neck and back pain. These practices are also promoted by the Chamber of Labor for Workers and Employees in Vienna, Austria (Kammer für Arbeiter und Angestellte für Wien), and Hungarian occupational health experts. In Thailand, a study was conducted on a series of relaxing exercises prescribed for office workers, incorporating complex eye, head, and neck movements [61]. Kolpakov and colleagues patented several eye health-focused movement and massage forms 36 years ago following their research [6,36,62,63]. These exercises (Figures S1–S17), which can be performed both at home and at work, aim to enhance overall body and eye blood circulation, thereby preserving eye health. Similarly, the lifestyle program developed by Japanese Katsuzō Nishi [6,64], which includes capillary exercises (Figures S18 and S19) targeting both body and eye health based on the mZalmanov principle [6,65], follows the same principles. A recent Japanese study also incorporated cognitive behavioral therapy-based exercises, including classic sports warm-ups, sit-ups, crunches, squats, arm and leg lifts, etc. [50]. Chinese eye exercises, based on massaging periocular anatomical points with the fingertips according to traditional Chinese medicine, aim to strengthen the eye muscles, improve blood circulation, and reduce eye fatigue [66]. Nevertheless, it proved effective for dry eye and asthenopic complaints observed during office work [57,67,68,69]. Overall, the authors believe that eye-relaxing office exercises targeting the entire body and head–neck region with active movement and muscle work could be beneficial for diabetic patients with a sedentary lifestyle to reduce the risk of DR [49,50,54,61]. It is important to highlight that no recommendations or studies have been made regarding some of these exercises for DR, but among these, Kolpakov et al. recommended and patented their gymnastics for preventing DR and enhancing retinal health [6].

#### 4.1.1. Bates–Schneider Method

The Bates–Schneider method, including ocular and visual training, as well as ocular yoga procedures, is based on the Bates technique, which is widely promoted by behavioral optometrists and visual therapists (Figures S20 and S21) [57,58]. These oculomotor muscle exercises should be performed together with appropriate lifestyle management and diet counseling based on international recommendations in order to improve the patients’ quality of life as holistically as possible [70]. For the proper functioning of the extraocular muscles, the visual trainer compiles the exercises in such a way that all eye muscles can be dynamically exercised, thereby increasing the muscles’ need for oxygen [70]. The exercises and professional experiences were published in 1891 and 1911 by William Horatio Bates, who attributed the origin of visual problems to mental stress, wrong gazing habits, and extraocular and facial muscle strain [57,71]. His technique emphasizes the original visual organ, the brain, instead of the eye, referring to the vision as mental process. Additionally, repetitive relaxation of the internal and external ocular muscles can enhance subjective visual function by relieving unnecessary mental and ocular stress. Meir Schneider is the founder of the self-healing method based on Bates’ visual exercises promoting active “body–mind work”. This method offers combinations of massage, physical exercises, visualization, breathing exercises, and a complete lifestyle program. Schneider believes that all patients can learn techniques to empower themselves to improve their own health, mostly focusing on rehabilitation [72]. The Bates–Schneider method is generally misinterpreted in the laymen and even scientific opinions because the original observations of Bates are different regarding visual performance than what the popular culture currently refers to. The application of this method in the refractive error’s viewpoint can be harmful without an ophthalmologist’s professional control.

The Bates–Schneider method can be useful for patients with visual impairment (such as DR) in increasing self-confidence and reducing their fears and insecurities associated with their eye disease. Integrating these techniques into a low vision rehabilitation approach can support patients with DR by improving their overall well-being and increasing focus on ocular health. However, it is crucial to note that the effectiveness of the Bates–Schneider method in treating refractive errors has been largely debunked by critics and scientific reviews, with no credible evidence supporting its use for DR management. Anecdotal evidence suggests that visual improvements in these cases are based on short-term blurry adaptation and perceptual learning rather than actual objective vision improvement. Therefore, further well-designed clinical studies are needed to provide objective scientific validation towards DR.

##### Sunning

Along with the skin of the human body, the eyes are the organs most exposed to ultraviolet radiation [73]. Sunning, a Bates visual exercise, means enjoying the light of the sun with closed eyes (to avoid retinal photodamage, e.g., solar maculopathy) and performing rhythmic movements of the head and hands (Figure S20B–D) [57,74]. From an ophthalmological point of view, sunning can be a method for slowing down the progression of myopia, but the scientific literature does not directly indicate that sunshine has a positive effect on DR. If the authors had to mention a possible positive outcome of the sunning method as a rehabilitation technique in relation to DR, it would be the following: Payne et al. assessed the significant relationship between insufficient serum vitamin D in the case of DR, especially in patients with proliferative DR, compared to a non-diabetic population [75]. There is some evidence suggesting that vitamin D, which is synthesized in the skin through exposure to sunlight, may have beneficial effects on diabetes and its complications. Vitamin D is known to reduce inflammation and oxidative stress, both of which are key factors in the development and progression of DR [76]. Adequate levels of vitamin D (and its oral supplementation) may help in maintaining better glycemic control and reducing the risk of complications associated with diabetes [76]. According to professional recommendation, approximately 5–30 min of direct sunlight on the face, arms, legs, or back twice weekly can provide the amount of vitamin D necessary to protect bones from the development of rachitis and/or osteomalacia. The consumption of 1800 IU (international unit) of vitamin D per day improves retinal blood flow [6,77]. Other sunlight-dependent biochemical pathways lead to the synthesis of alpha melanocyte-stimulating hormone, calcitonin gene-related peptide, neuropeptide substance P, and endorphins; the latter explains the psychologically beneficial effect of sunbathing [73]. Based on the above, the Mediterranean lifestyle takes on a new meaning; in addition to high-quality red wine with a high resveratrol content and a Mediterranean diet rich in antioxidants, a sufficient amount of sunlight is also considered a protective factor [46,78,79]. Emphasis must be placed on a moderate healthy amount of sunlight exposure: according to Simó and Hernández, this method maintains vitamin D biosynthesis, but sunlight exposure for ≥5 h a day is significantly associated with an increased risk of DR [80,81].

##### Swaying and Swinging

Swaying and swinging are underestimated and neglected components of the Bates–Schneider method [57]. During the rhythmic swinging of the head or the whole body [57,74], the eyes are forced to focus on a specific target object that is aligned stationarily on the fovea (Figure S2). Visual information from the environment is gathered through these quick eye movements, which consist of a series of saccades and fixations [82]. Saccadic movements are also important in the abovementioned “sunning” technique. Fixation saccade training as a gait rehabilitation strategy can be an important therapeutic option to improve movement coordination of patients with progressive visual loss, including those with DR. The applied elements of this rehabilitation strategy are balance training, foot stepping, and eye movement exercises [82]. Based on this hypothesis, the Bates method of swaying–swinging may be incorporated into low vision rehabilitation to lower the risk of falls and difficulties of a low quality of life. These exercises can help to enhance the coordination and balance of patients, which is crucial for those with visual impairments. Incorporating such activities into the route of individuals with DR may aid in maintaining better ocular health and overall well-being, supporting the principles of lifestyle medicine.

### 4.2. General and Ocular Yoga

Yoga (Sanskrit term meaning “to connect”) has become increasingly popular, with its regular practice doubled within a 10-year period, increasing from 0.46% in 1999 to 1.11% in 2008 [83]. According to a 2022 report from the National Health Interview Survey, 16.9% of adults aged 18 and older in the United States practiced yoga in the past 12 months [84]. The principal elements of yoga are asanas and mudras (leg and hand positions) with breathing exercises and meditation [83]. Considering the fact that vision loss and psychosocial emotional responses go together, relaxation, psychotherapy, or other stress reduction programs should be helpful in reducing the impact of low vision [85]. The regular practice of yoga results in increased alpha and theta brain activity, thus alleviating autonomic functions [83]. Yoga has been mentioned to help in managing type 2 diabetes mellitus, which can indirectly benefit DR by improving glycemic control, reducing stress, and enhancing overall health. Improved glycemic control can help prevent or slow the progression of DR [86]. Nathani and Nanduri reported in a case study that a patient with DR experienced significant improvement in vision and overall diabetes management after following Yoga Prana Vidya healing protocols. The patient showed reduced blurriness in vision and better control of diabetes [87]; however, since this is evidence from a single case, its scientific power is very low. Yogic ocular exercises, including extraocular muscle exercise (palming, blinking, gazing in nine directions, focusing on near and distant points, accommodative exercises, etc.), Trāṭaka Kriya, and 45 min of daily meditation, also significantly reduce intraocular pressure and anxiety.

Ocular yoga, an advanced version of the Bates method stemming from Indian Ayurvedic medicine, includes exercises such as circular eye movements, focusing on objects at different distances, and resting the eyes [88,89]. A specific subtype is the meditative practice Trāṭaka kriya (yogic gazing) [88], which has been studied as a complementary treatment for glaucoma patients, but its role in DR is unknown. Ocular yoga exercises also affect the average retinal thickness of the macula in a favorable way and increase oxygen saturation in the blood through deep breathing [89]. Kumar et al. reported a 3-month Ayurvedic treatment protocol containing Rasayana yoga aimed at ameliorating DR. According to their analysis, they achieved the following primary outcome: the trial group showed statistically significant improvement in superficial and dot-blot retinal hemorrhages, hard exudates, and visual acuity. However, the secondary outcome, hypoglycemia, was not statistically significant compared to the control group [90]. According to the AAO’s recommendations, patients with known associated primary or secondary glaucoma or ocular hypertension should be advised against practicing yoga exercises with 10 head-down positions (Śīrṣāsana, Adho Mukha Śvānāsana, Uttānāsana, Halāsana, Viparīta Karaṇī, Kākāsana, Vṛścikāsana, Adho-mukha-vṛksāsana, Piñcha-mayūrāsana, and Sarvāṅgāsana), which are associated with a transient 2-fold increase in intraocular pressure in glaucomatous and healthy eyes. Regular practicing of the previously described positions caused visual field progression [91,92]. Further limitations in yogic practice are that diabetic patients (similarly to myopic patients) have been shown to have an altered composition of their vitreous body, which may predispose them to vitreous degeneration or syneresis. During yoga exercises, postural changes, especially when transitioning from an upright position to a head-down position and vice versa, may cause abrupt shifts in the vitreous gel. Vitreous hemorrhage may be seen in acute posterior vitreous detachment due to the spontaneous rupture of small retinal capillaries [93]. Not only can vitreous shear forces cause retinopathy, but the Valsalva mechanism induced by a closed glottis during breathing exercises can also contribute to this condition. Recently, Kaushal et al. documented the first case of Valsalva retinopathy resulting from 30 min of daily Anuloma pranayama (a type of Hatha yoga) over a one-month period [94]. The retinas of patients with DR are particularly susceptible to pressure changes associated with the Valsalva maneuver. Altogether, yoga is considered a safe and effective complementary practice affecting DR. However, based on the AAO’s recommendations, DR patients should disclose their activity to their treating ophthalmologist and other specialists evaluating the risks and benefits regarding their ocular condition.

## 5. Psychosomatic Correlations of Eye Diseases

Patients with severe vision loss from DR face significant challenges when performing daily tasks, driving, and moving [85,95], which affects their psychosocial well-being. While there is a wide range of studies in the literature on the quality-of-life assessment of, e.g., DR patients, there is less information about exact methods to improve it. Educating the patient about the disease and encouraging two-way communication between the patient and physician are known to reduce anxiety and stress. Inadequate information and uncertainty due to fear-inducing diagnosis and prognosis also make healthcare personnel responsible [85,95]. Important parts of the treatment process are empathy and taking the time to explain the illness to the patients, who can often become isolated without much understanding from medical professionals and/or their relatives. Later, patients feel that their disease is untreatable, which leads to a decrease in compliance and discontinuation of treatment [96]. It is important as therapists to explain to patients that their ocular disease is chronic and that the severity of symptoms may fluctuate depending on the body’s internal conditions and reactions to environmental conditions [96]. Even though the eye is a relatively small organ of the human body, the term “sick eye in a sick body” syndrome is increasingly appropriate, as Dada et al. states, as it can be associated with disorders of the cardiovascular, central nervous, and endocrine systems. Excessive stress exposure of the brain to glucocorticosteroids can become toxic to neurons and even retinal tissue [46,85]. Psychological stress can also activate inflammatory responses through the neuronal activation of signaling pathways, which ensures increased nuclear factor kappa B (NF-κB) production [46,85]. In glaucomatous, diabetic, and healthy eyes, mental stress is associated with an increase in IOP and vasoregulatory disturbances caused by glucocorticoids, proinflammatory cytokines, and endothelin-1 [46,85]. Tumor necrosis factor alpha (TNF-α) and interleukin 6 (IL-6) levels also increase in the aqueous humor. These factors can all contribute to the loss of metabolic control. Thus, if metabolic shift slows down, degenerative processes gain ground, causing diseases of the retina [46,85]. These processes can be further aggravated by multiple stressors, and therefore, it is necessary for ophthalmologists and other health professionals to understand the usefulness of stress-reducing therapies in ophthalmology, e.g., for diabetic patients [97]. Complementary therapies such as yoga, meditation, autogenic training, music therapy, and psychotherapy support have been shown to reduce stress—related to vision loss and fear of blindness—and improve quality of life [85,95]. Depression and suicide rates are notably higher among individuals with DR compared to the general population. According to a study by Bao et al., the prevalence of depression in patients with moderate to severe non-proliferative DR or proliferative DR was significantly higher (14.3%) compared to those with mild retinopathy or no retinopathy (6.9% and 7.0%, respectively) [52]. Additionally, a study highlighted by Ha et al. found that individuals with sight-threatening eye diseases, including DR, had a higher incidence rate ratio of suicide compared to those without such diagnoses. Specifically, DR was associated with the highest suicide deaths among sight-threatening eye diseases, accounting for 57% of cases [53].

The holistic model of healthcare emphasizes the integration of physical, mental, emotional, and spiritual needs of patients. This model suggests that addressing these aspects in a balanced manner can lead to better health outcomes [98]. Research by Sridhar showed that spirituality and religion can play a significant role in coping with diabetes. Spiritual practices and beliefs can help manage the emotional and psychological stresses associated with diabetes, contributing to overall well-being [99]. Another study highlighted the significant emotional and social strain experienced by individuals with DR. This strain can affect their overall health and well-being, indicating the importance of addressing emotional and social factors in managing the condition [100]. Furthermore, spiritual intelligence and mindfulness can positively influence the mental well-being of individuals with diabetes. These factors can help manage emotional dysregulation and depression, which are common in diabetes [101,102]. These findings underscore the importance of comprehensive care that includes mental health support for individuals with DR to address the increased risk of depression and suicide.

### 5.1. Mind–Body Therapies (Meditation and Visualization)

Mindfulness-Based Stress Reduction (MBSR) is a structured program that combines mindfulness meditation and gentle yoga. Developed by Jon Kabat-Zinn, MBSR aims to cultivate nonjudgmental awareness of the present moment, which can help individuals manage stress and improve overall well-being [102]. For people with diabetes, MBSR has been shown to reduce stress, improve glycemic control, and enhance quality of life [102]. By reducing stress and promoting relaxation, MBSR may indirectly benefit eye health and help manage DR. A bibliometric study by Jiang et al. highlighted the increasing interest in mindfulness interventions for diabetes. While the primary focus is on mental health and metabolic control, these interventions could potentially benefit overall eye health and reduce the risk of complications like DR [103]. Other mind–body therapies, including meditation, yoga, and other relaxation techniques, are promising options for managing diabetes [104]. These therapies aim to counteract the stress response and promote a state of relaxation, which can help regulate cortisol and other stress hormones [104]. Chronic stress is known to negatively impact blood glucose control and contribute to the development of diabetic complications [6,104]. By reducing stress and improving metabolic control, mind–body therapies may help prevent or slow the progression of DR. Meditation and yoga are traditional practices that focus on mental and physical well-being. Meditation involves focused attention and diaphragmatic breathing, which can help reduce stress and improve emotional health [104].

In conclusion, the direct impacts of meditation, yoga, and mindfulness on DR require further research. Incorporating these complementary therapies into a comprehensive diabetes management plan may help improve overall health and potentially benefit eye health in individuals with DR.

### 5.2. Effects of Eye Eurhythmy on Diabetic Retinopathy

Eurhythmy therapy [Ancient Greek: εὖ (good) + ῥυθμός (regular, symmetric motion)] is an active exercise of anthroposophic medicine, introduced by the Austro-Hungarian Rudolf Steiner in 1911 [105,106,107]. According to the principles of anthroposophy, a person’s physical, emotional, and spiritual–individual levels interact harmoniously, and their imbalance is a characteristic of illness. Therefore, the central idea of eurhythmy is to restore the balance of health with meditative movement exercises [108]. Speech movements are transposed into exercises, targeting the patient’s self-expression and self-healing abilities. In general, eurhythmy improves the breathing pattern and posture, strengthens muscle tone, and increases physical vitality, so it is particularly beneficial in the complementary treatment of neurological locomotor diseases [105,106,107]. The question of whether eurhythmy cannot also be used for eye diseases due to the neural connection may arise. The first steps toward the development of eurhythmy exercises for ophthalmic diseases (in German, Augeneurhythmie) was first invented and standardized by Ilse Knauer between 1938 and 1971 [107]. Margret Thiersch reports on the symbolism of eurythmic body movements, which can basically help the patient to perceive and visualize their eyes and vision more consciously [106]. Paul Blok und Ralf Brukart advise patients with DR to perform therapeutic eurhythmy exercises with the “D”, “T”, “I”, “M”, and “S” sound series daily in addition to relaxation techniques and breathing exercises. According to the principles of eurhythmy and with the addition of the authors’ opinion, “D” symbolizes the response to external stimuli, and its downward movement imitates the reduction in blood sugar levels. The “T” sequence, with its analogy of moving from top to bottom and from outside to inside, models cellular processes, such as glucose uptake by the cell through the GLUT4 (Glucose Transporter Type 4) transporter. The stretching exercises of the “I” sequence focus on the patient’s individuality and symbolize self-reflection, projecting the direction of vision. The “M” sequence represents the bidirectional metabolic processes occurring in the human eye and body. In terms of vision, it serves to raise awareness of DR and the acceptance of sometimes incurable conditions, alongside the “groping” in darkness associated with blindness and low vision. The complexity of the “S” sequence not only illustrates the dynamism of the retina and the body’s blood circulation but also serves to relax the patient, as order emerges from chaos [106,109]. Anger suggests that eurythmy can serve as a secondary prevention intervention for diabetic patients [110]. However, the psycho-philosophical concepts underlying the two types of diabetes differ: In type 2 diabetes, the condition is attributed to a “weakened Ego”. In type 1 diabetes, the “Ego” has withdrawn, leading to an autoimmune response [110]. Hilgard suggested eurhythmy as complementary therapy among children and adolescents with type 1 diabetes. According to her recommendations, the cornerstones of childhood diabetes management are glycemic control with insulin substitution and antidiabetics, which should be completed with eurhythmy, art therapy, and individual psychological care [111]. During eurhythmy, participants engage in skill-based activities and rhythmic exercises. The primary objective is to cultivate lifelong somatic awareness in adolescents with type 1 diabetes, ensuring meticulous regulation of glucose homeostasis [111]. In the context of DR, direct supporting evidence is limited and warrants further research; although eye eurhythmy provides a tool for a patient in accepting their disease and reducing anxiety, there is no available placebo-controlled clinical study. After eurhythmy treatment, increased autonomic nervous system-associated stress adaptation can be expected, as well as general improvement in cardiovascular parameters, ultimately leading to a better quality of life. Adult and pediatric patients generally report good cooperation and satisfaction without any known documented side effects [112]. Based on several clinical results, it significantly improves the sense of self-competence in patients with chronic diseases; improves posture and breathing and heart rate patterns; and strengthens muscle tone [108,113].

### 5.3. Shinrin-Yoku

The Japanese term Shinrin-Yoku (kanji: 森林浴; “forest bathing”) was first introduced by the Ministry of Agriculture, Forestry, and Fisheries of Japan in 1982 [114]. Studies have been conducted to investigate the effects of forest environments on human physical and mental health since 2004 [115]. Recently, Li established the term “Forest Medicine” in 2022, referring to a potential healing and rehabilitation technique with a “prevention is better than cure” lifestyle approach [114,115]. Dynamic forest bathing refers to patients participating in forest environment interventions (e.g., alpinism, hiking, yoga, etc.) and effectively enhances the activity of the parasympathetic nervous system while reducing the activity of the sympathetic nervous system. Other physiologic benefits are the increase in human natural killer cells and the intracellular levels of anti-cancer proteins, as well as reductions in blood pressure and heart rate [114]. From an ophthalmologic point of view, forest landscapes with naturally occurring colors (green, yellow, and red) potentially stimulate the visual pathway and photoreceptors. Everyday technostress (electric devices, artificial lighting, etc.) affecting the visual system ultimately leads to mental stress, anxiety, depression, computer vision syndrome (headaches, mental fatigue, eye, and neck strain), and insomnia [115]. Ohtsuka et al. found that Shinrin-Yoku significantly decreased blood glucose levels in non-insulin-dependent diabetic patients. The study involved 87 diabetic patients who participated in forest bathing sessions for over six years. The results showed a significant reduction in mean blood glucose levels and glycated hemoglobin (HbA1c) after the forest bathing sessions [116]. These findings suggest that Shinrin-Yoku can be an effective complementary therapy for managing blood glucose levels in diabetic patients, likely due to the combined effects of physical activity, stress reduction, and the natural environment. According to the studies of Marigold and Hollands, it can be useful to guide patients on a therapeutic tour (e.g., Shinrin-Yoku and nature therapy) for rehabilitation purposes, as walking on uneven ground or terrain requires visual information to guide foot placement, and anxiety is reduced in a green environment [82]. Due to possible indirect neuroprotective effects, it may have complementary benefits for patients with DR by reducing stress, improving mental health, and potentially enhancing overall ocular health. Although more scientific evidence needs to be collected and evaluated, incorporating Shinrin-Yoku into a lifestyle medicine approach could support traditional treatments for DR.

## 6. Clinical Considerations and Recommendations

Patients with DR or glaucoma suffer from greater visual field loss, altered color vision perception, and decreased stereoacuity due to the apoptosis and autophagy of retinal ganglion cells, which all affect activities of daily life [46,117,118].

Eye movement disorders caused by strabismus and eye muscle paralysis of various origins (e.g., diabetic neuropathy, etc.) [42,119] cause diplopia and other visual disturbances in addition to esthetic problems. Vision deterioration caused by advanced DR and post-operative complications, e.g., progressive proliferative diabetic vitreoretinopathy even after successful pars plana vitrectomy, makes patients visually impaired with an ever-decreasing quality of life.

**Figure 1:**
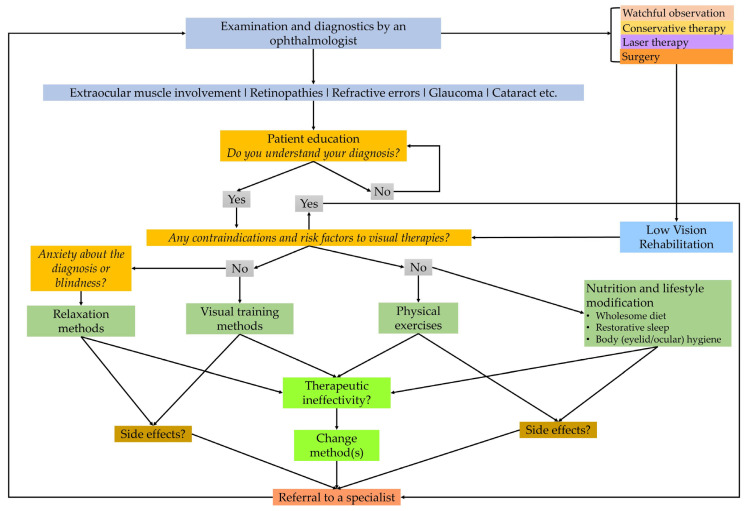
A flowchart of clinical steps in integrative medical care helping ophthalmologists, visual therapists, and behavioral optometrists in eye-related lifestyle interventions. The chart emphasizes the importance of correct diagnosis and strict medical treatments based on the recommendations of a specialist ophthalmologist (self-edited).

While in the case of the former diseases, the functions of visual perception and facial and eye muscles would be partially or optimally fully restored, in the latter case, the aim would be to develop functional vision that can be developed even with “impaired vision”. Integrating lifestyle medicine and low vision rehabilitation principles, such as a balanced diet, regular physical activity, stress management, and proper eye hygiene, into patients’ care plan can support overall eye health and improve quality of life (Figure 1). Current recommendations for DR management include strict control of blood sugar levels, blood pressure, and cholesterol, along with regular eye examinations and timely treatment interventions or medications, laser therapy, and surgical interventions. These are supported by a wealth of evidence for their efficacy [46]. It is also essential for healthcare providers to clearly evaluate the ophthalmic conditions of their patients to select complementary therapy or even discourage certain practices. Patients with DR may have other ocular comorbidities like glaucoma or high-grade myopia, increasing the risk of vitreous liquefaction, vitreous hemorrhage, tractional retinal tear, or retinal detachment [6,93]. The tractional movement of the vitreous body causes the spontaneous rupture of the small capillaries of the retina, so, due to the aforementioned risks, according to the authors’ professional opinion, Kolpakov’s gymnastics, Nishi’s capillary exercises, and Bates’ swaying–swinging exercises are not recommended in order to avoid vitreoretinal injuries [6,93,120]. Prevention, information, and advice are also provided by other specialists, e.g., opticians, optometrists, and rehabilitation visual trainers, to facilitate the work of ophthalmologists [121]. Therapies must be regularly checked by an ophthalmologist and modified if necessary. Although longer treatment times and patience are required, these therapies significantly improve the quality of life [96].

**Figure 2:**
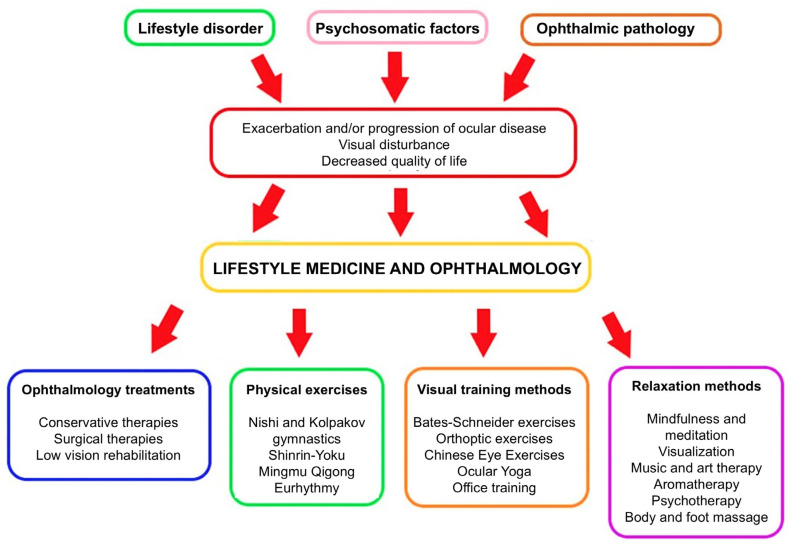
The role of lifestyle medicine in the system of ophthalmology treatments. If a specialist has identified multifactorial causes of the patient’s eye disease impairing quality of life, a therapeutic decision can be made in addition to the professional treatment of the pathophysiological cause, as well as personalized complementary therapy involving lifestyle change (self-edited). The red arrow in the flowchart represents a transition point where the negative outcomes—exacerbation or progression of ocular disease, visual disturbance, and decreased quality of life—lead to the implementation of lifestyle medicine and ophthalmology interventions.

A limitation of this study is the limited scope of direct evidence in the scientific literature. There are few or no high-impact, peer-reviewed studies directly correlating specific complementary therapies with measurable improvements in DR outcomes. Most of the presented practices could indirectly or directly improve the systemic conditions caused by DR and diabetes, reducing their progression, and this study can help initiate scientific dialog and promote evidence and research in this field in the future. Future research may provide more insights into their potential roles. For those interested in complementary therapies, it is essential to consult with healthcare professionals to ensure any adjunctive treatments are safe and do not interfere with conventional treatments. Prevention, information, and advice are also provided by other specialists, e.g., opticians, optometrists, and rehabilitation visual trainers, to facilitate the work of ophthalmologists [121]. Therapies must be regularly checked by an ophthalmologist and modified if necessary (Figure 2).

## 7. Conclusions

Low vision exercises and aids can help patients make the most of their remaining vision, improving their ability to perform everyday tasks and reducing the impact of vision loss on their lives. By providing tools and techniques to enhance visual function, low vision aids and exercises enable patients to maintain a higher level of independence. This can lead to improved self-esteem and a greater sense of control over their condition. Vision loss can be associated with an increased risk of depression and anxiety. By improving visual function and reducing the challenges associated with vision impairment, low vision aids and exercises can contribute to better mental health and overall well-being. In conclusion, low vision exercises and aids play a crucial role in the comprehensive management of DR. These interventions help patients maximize their remaining vision, maintain independence, and improve their quality of life. Incorporating these strategies into the care plan for DR patients can lead to better outcomes and a more positive outlook on managing their condition.

## Figures and Tables

**Table 1:** Classification of low vision rehabilitation modalities.

Low Vision Rehabilitation Modalities ^1^	
Optical aids	For near and intermediate activities
	For distant activities
Non-optical aids	Environmental modifications
	Digital devices
	Non-electric aids
	Animal-assisted therapy
Psychotherapy	Psychological consultation
	Mind–body practices
Neurofunctional rehabilitation	Biofeedback training
	Vision therapy
Prostheses	Implantable intraocular devices (telescopic IOL ^2^, bionic retina, etc.)
	Extraocular devices (orbital and ocular prostheses, prosthetic contact lenses, etc.)
Cellular and gene therapy	Stem cell therapy

^1^ Self-edited table adapted from multiple sources: Cooke et al. (2001), Muhsin et al. (2024), Németh et al. (2024), O’Loughlin et al. (2024), and Vingolo et al. (2015) [16,17,18,19,20]. ^2^ IOL: intraocular lens.

## Data Availability

No new data were created or analyzed in this study. Data sharing is not applicable to this article.

## Supplementary Materials

The following supporting information can be downloaded at: https://www.mdpi.com/article/10.3390/life15060857/s1. Figures S1–S7: Kolpakov’s industrial (wrokplace) gymnastics; Figures S8–S17: Kolpakov’s hygienic (domestic) gymnastics; Figures S18–S19: Katsuzo Nishi’s workout; Figures S20–S21: Bates-Schneider exercises.

## Abbreviations

AAO	American Academy of Ophthalmology
AHA	American Heart Association
BDNF	Brain-Derived Neurotrophic Factor
DR	Diabetic Retinopathy
GLUT4	Glucose Transporter Type 4
IL	Interleukin
IU	International Unit
MBSR	Mindfulness-Based Stress Reduction
NF-κB	Nuclear factor kappa B
TNF-α	Tumor Necrosis Factor Alpha (α)
WHO	World Health Organization
