# Supplementary material for: Low Vision Rehabilitation and Eye Exercises: A Comprehensive Guide to Tertiary Prevention of Diabetic Retinopathy
Source: Life (Basel). 2025 May 26;15(6):857. doi: 10.3390/life15060857 (PMC12194327; doi:10.3390/life15060857)
Supplement: Supplementary file 1 [file life-15-00857-s001.zip › life-3565923-supplementary.pdf]

Supplementary material

KOLPAKOV'S INDUSTRIAL (WORKPLACE) GYMNASTICS

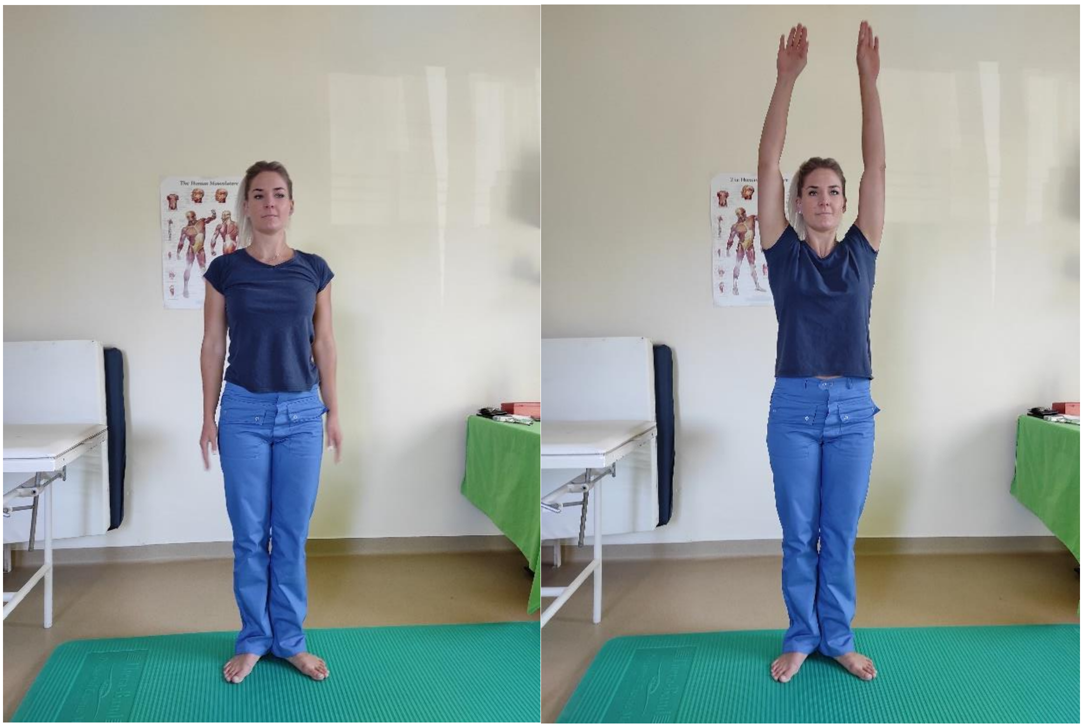

Figure S1. Primary position: standing on legs, heels touching together, toes apart, arms next to the thighs. Counting 1-2: rise on the toes, raising the arms in circular movement, stretching the body. Counting to 3-4: return to the starting position. Repetition: 4 times.

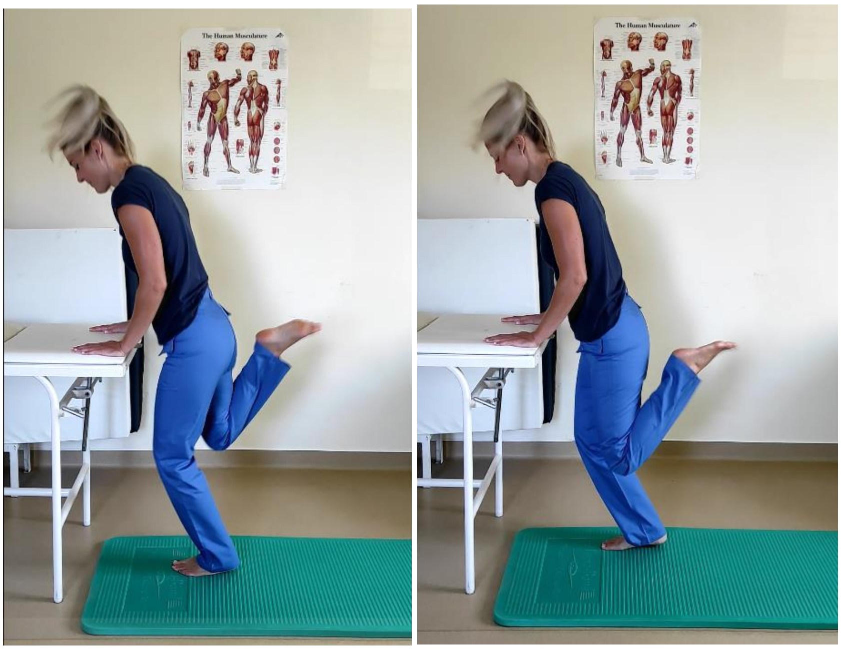

Figure S2. Standing at the desk, tilt the torso forward, lean on the table with the hands. Counting 1-2: alternate the legs. Duration: 1-2 minutes.

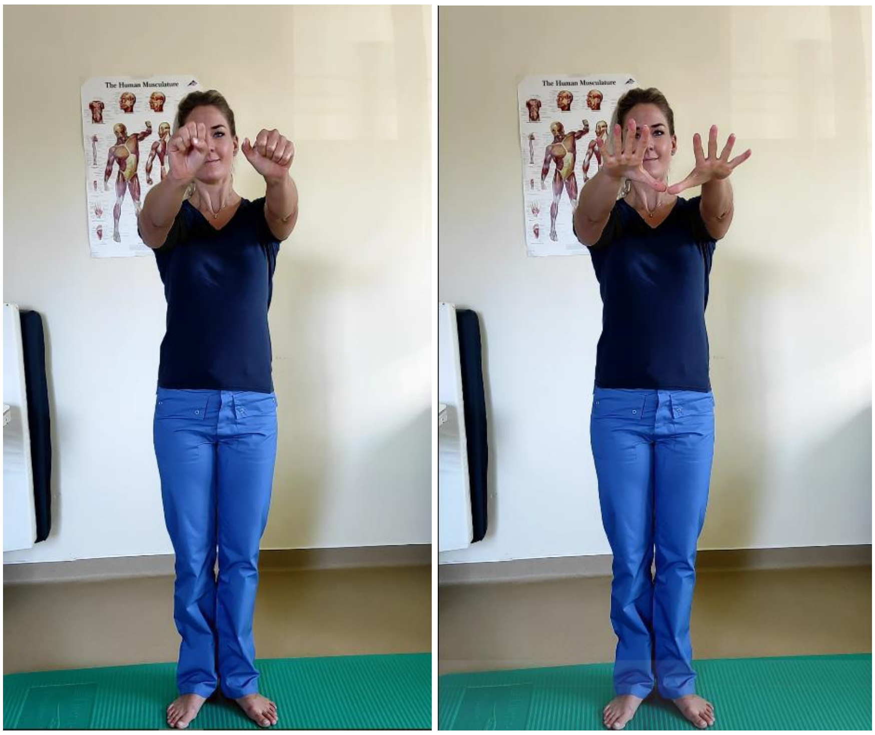

Figure S3. Place forward the hands from the chest. Counting 1-2: squeeze and extend alternately the fingers ("Fingerspritz"). Repetition: 15-20 times.

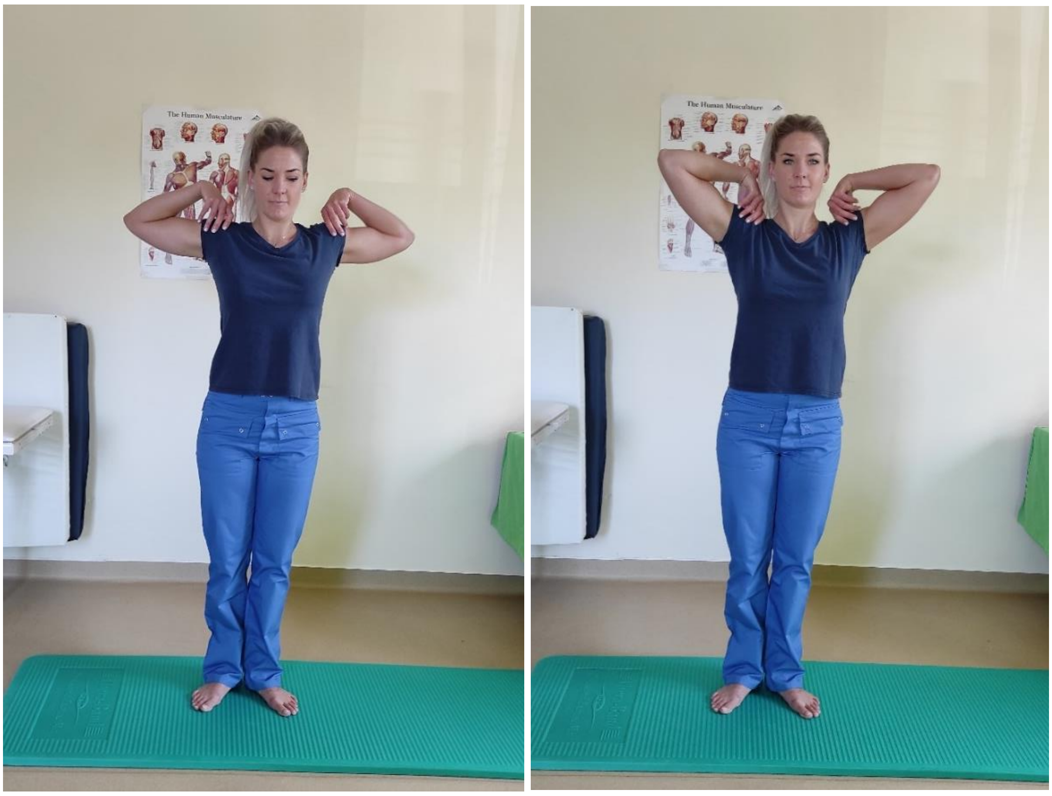

Figure S4. Primary: standing on legs, heels touching together, toes apart, arms next to the thighs. Bend the arms, hands touching the shoulders. Counting 1-4: circular movements forward, counting 5-8: circular movements backwards. Repetition: 15-20 times. fifteen to twenty times.

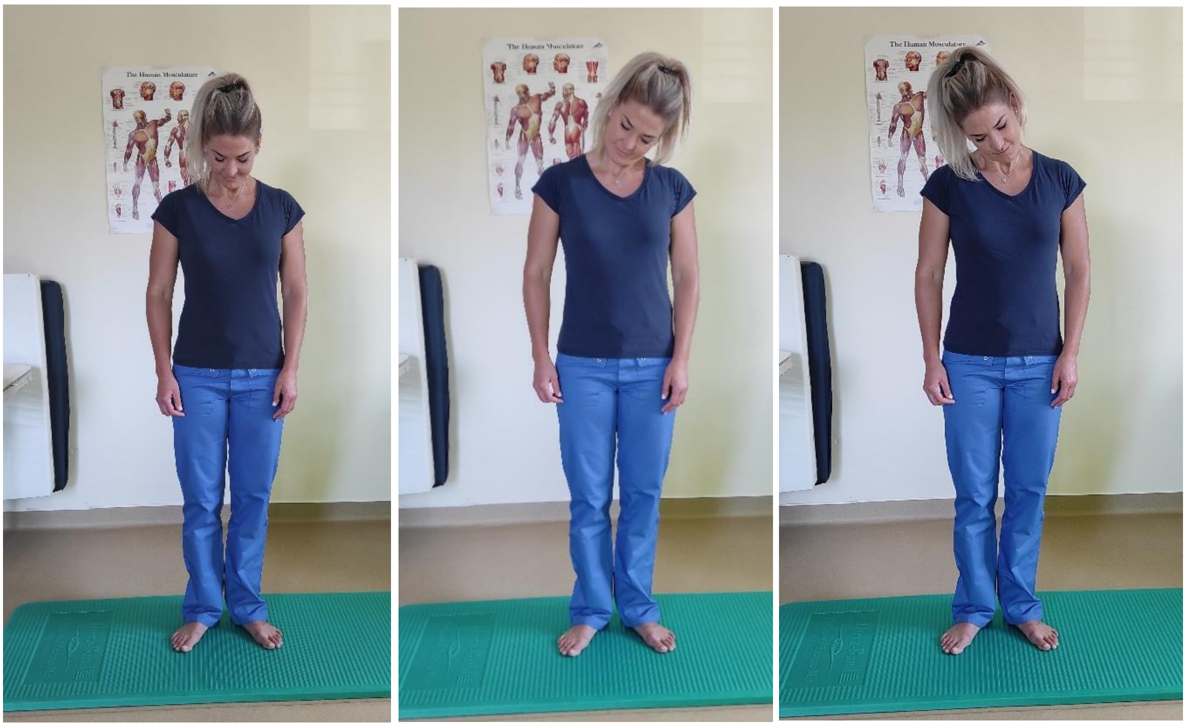

Figure S5. Primary position: standing on legs, heels touching together, toes apart, arms next to the thighs. Count 1: Touch the thorax with the chin, as possible. Count 2: Bend the head to the left shoulder. Count 3: The chin stays on the chest. Count 4: straighten the head in the primary position. Counting 5-8: the same movements in the direction of the right shoulder. Repetition: 5-10 times on both sides.

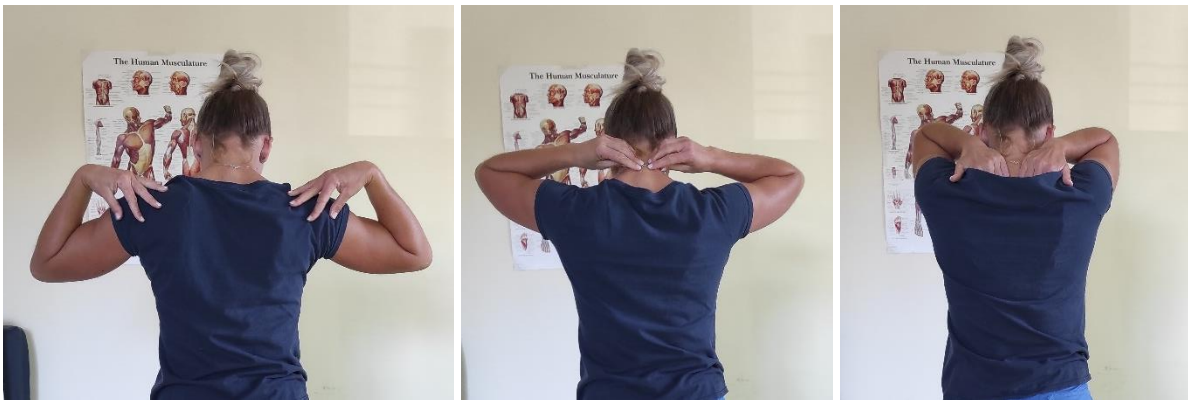

Figure S6. Self-massage with both hands on the posterior neck region and shoulders from the bottom up. Repetition: 3-4 times.

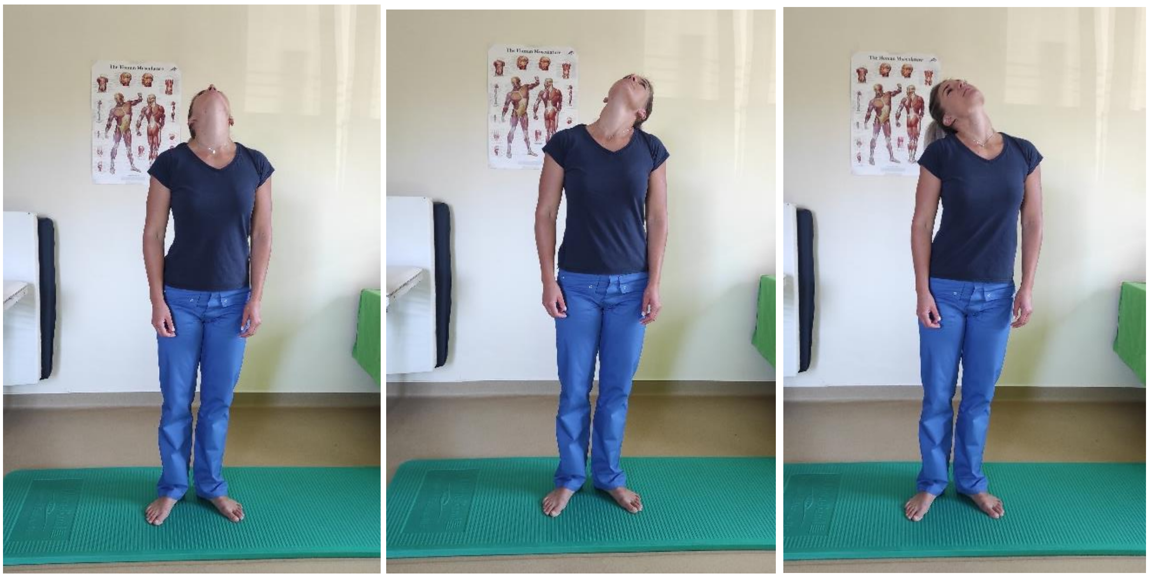

Figure S7. Primary position: standing on legs, heels touching together, toes apart, arms next to the thighs. Look up, bend the head backwards. Count 1: Keep bending the head to the left. Count 2: Straighten it. Count 3-4: The same to the right side. Repetition: 5-10 times on each side.

KOLPAKOV'S HYGIENIC (DOMESTIC) GYMNASTICS

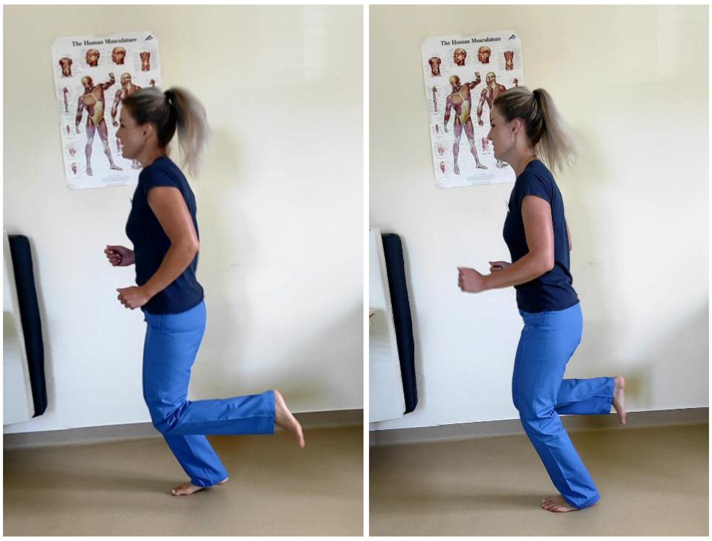

Figure S8. Walking and/or running for 3-5 minutes. Resting intervals are 10-20 seconds between each workout.

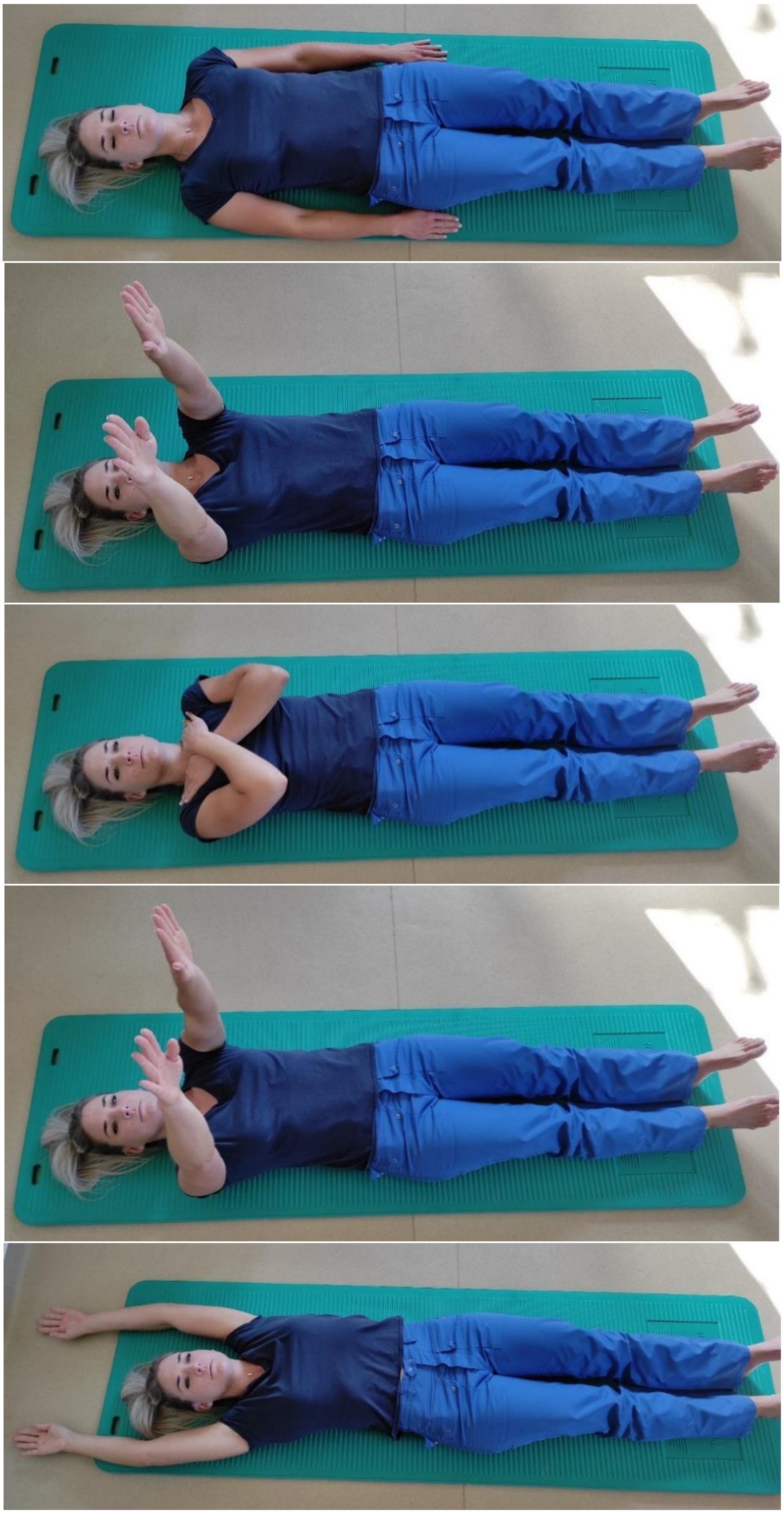

Figure S9. Primary position: lie down on the carpet/mat on the back, place the arms next to the thighs. Count 1: raise the arms forward to the chest. Count 2: lower the arms, crossing in front of the chest. Count 3: extend the arms in front of the chest. Count 4: raise the arms above the head. Counts 5-8: reversion of the steps.

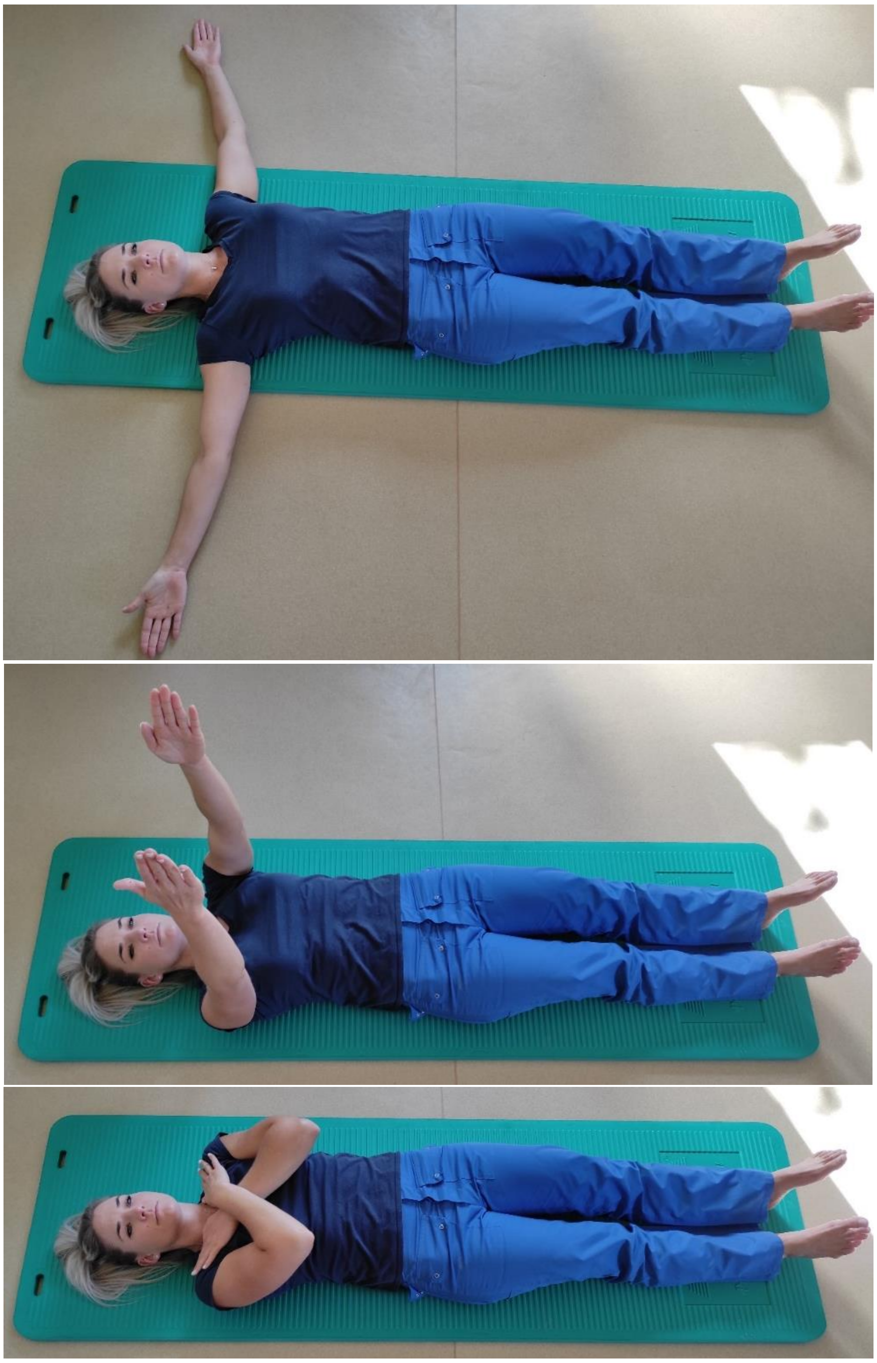

Figure S10. Primary position: lying on the back, stretching the arms chest wide with palms up. Count 1: raise the arms in front of the chest. Count 2: lower the arms, crossing in front of the chest. Count 3-4: raise the arms from the chest forward and place them in the starting position for four.

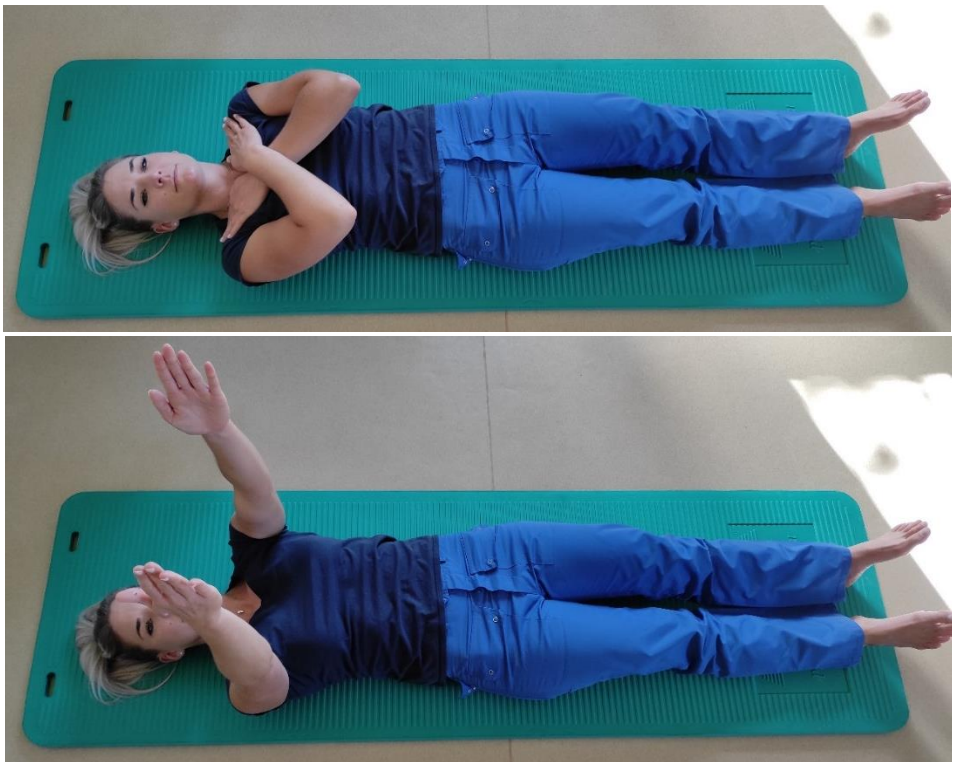

Figure S11. Stay lying on the back, put the hands on the chest. Count 1: raise the arms in front of the chest. Count 2: lower the arms back on the chest.

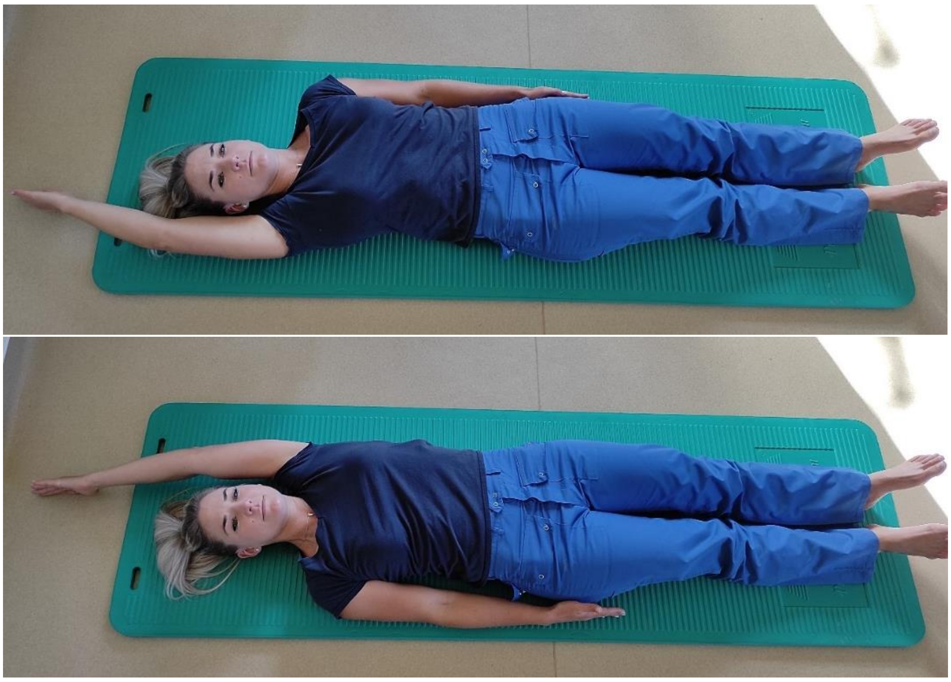

Figure S12. Lying on the back, alternate the arms up to the head counting 1-2.

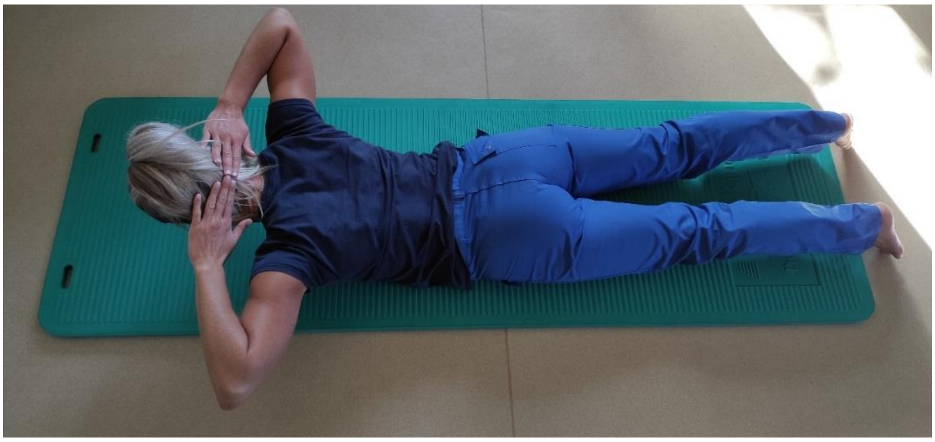

Figure S13. 6. Turn on the stomach with hands clasping over the posterior neck area. Raise gradually the upper part of the torso and stay in this position while counting to 3, then return to the starting position for 4.

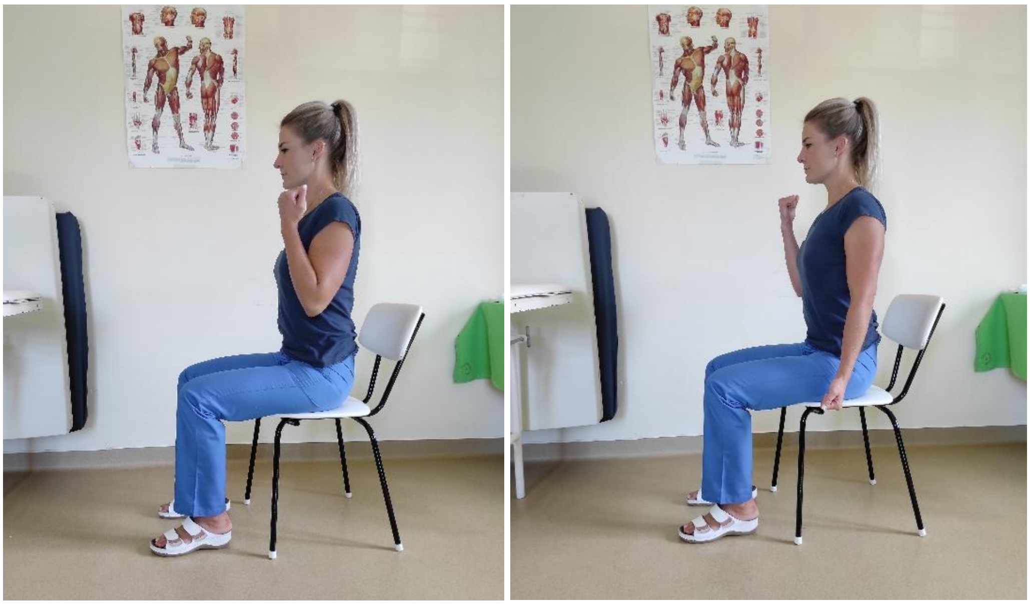

Figure S14. Primary position: Sitting on a chair, knees should be perpendicular to the ground, the arms should be lowered. Counting 1-2: bending and extending the arms (similar to weightlifting without dumbbells).

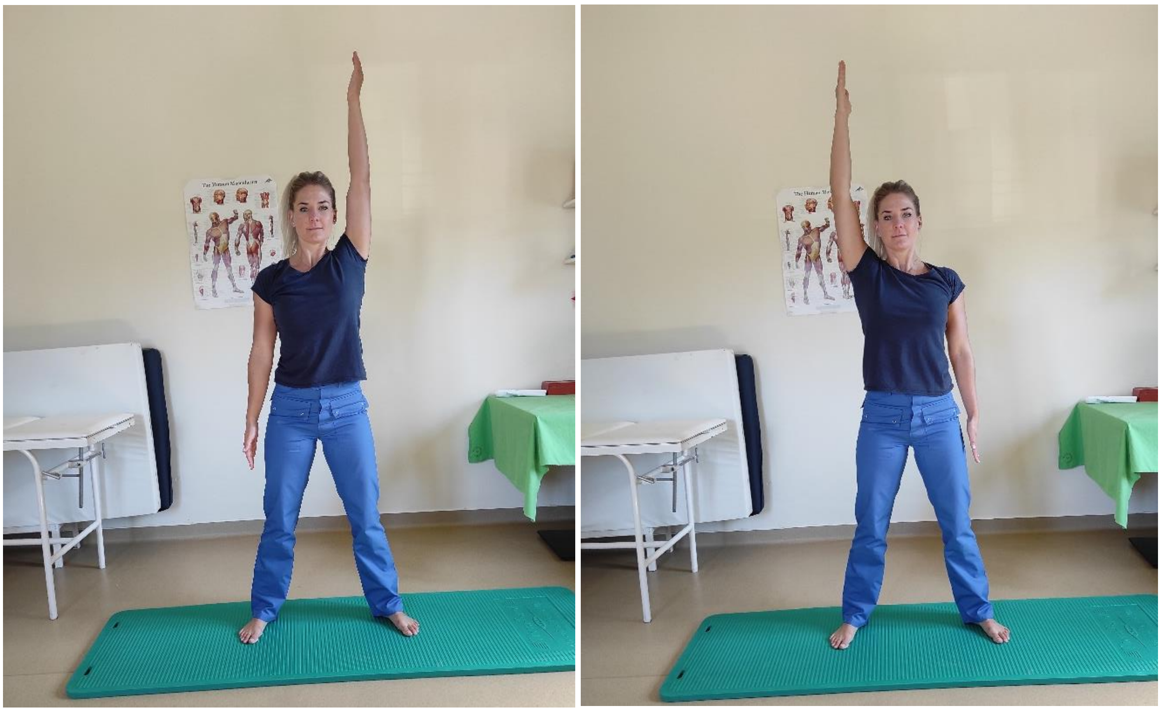

Figure S12. Primary position: standing with legs spread (feet shoulder-width) apart. Counting 1-2: alternate the up-down positions of the arms.

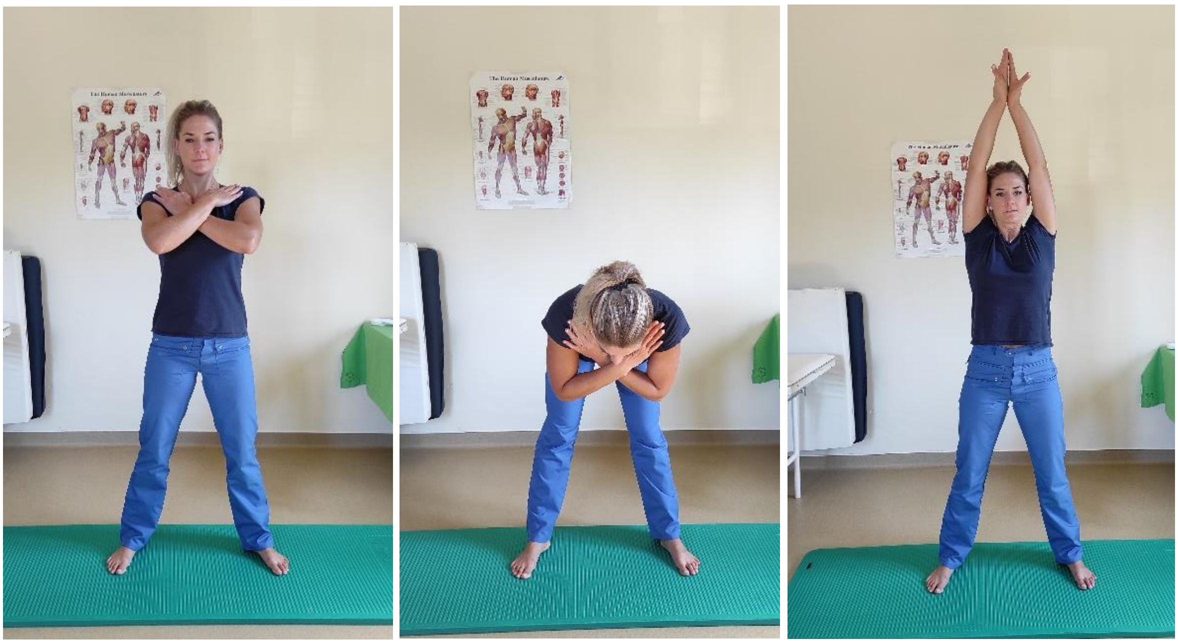

Figure S13. Primary position: standing with legs spread (feet shoulder-width) apart. Count 1: Tilt forward the body with the hands on your chest. Raising the upper body through the long arm stretching laterally. Touch the back of the hands above the head as an endpoint. Count 2: Return to the primary position.

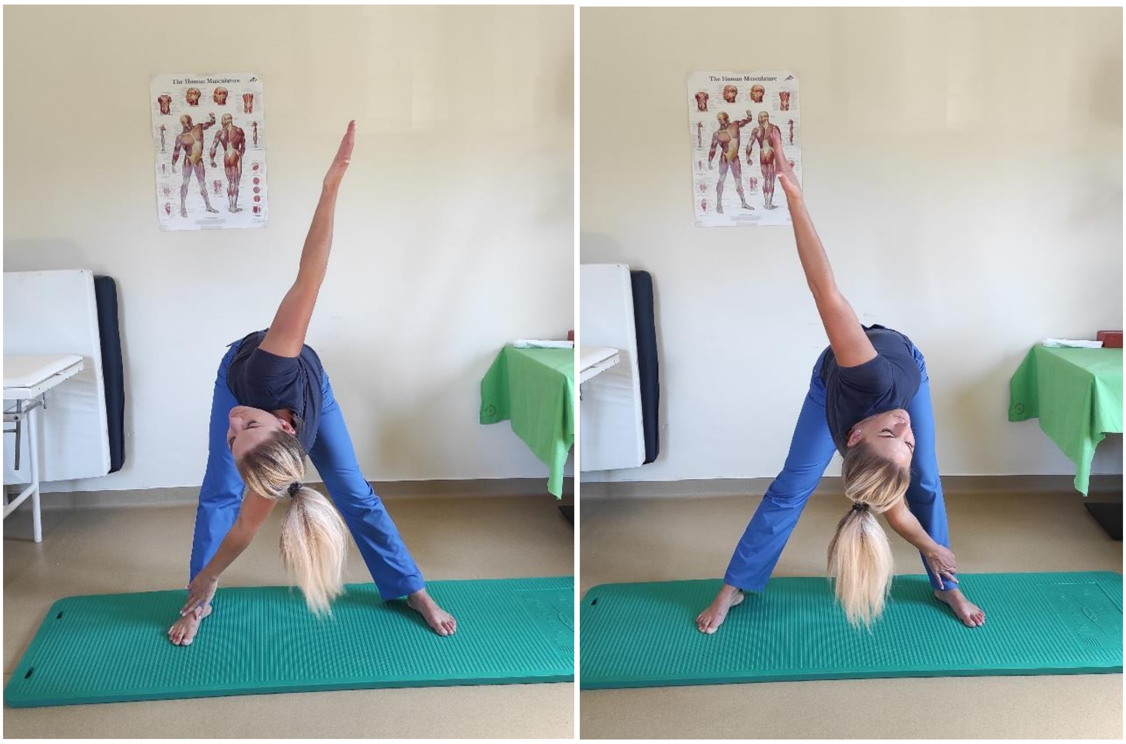

Figure S17. Primary position: standing with legs spread (feet shoulder-width) apart. Body is tilted forward, arms to the sides. Count 1-2: Alternating touch of toes with opposite hands ("Windmill").

KATSUZŌ NISHI'S WORKOUTS

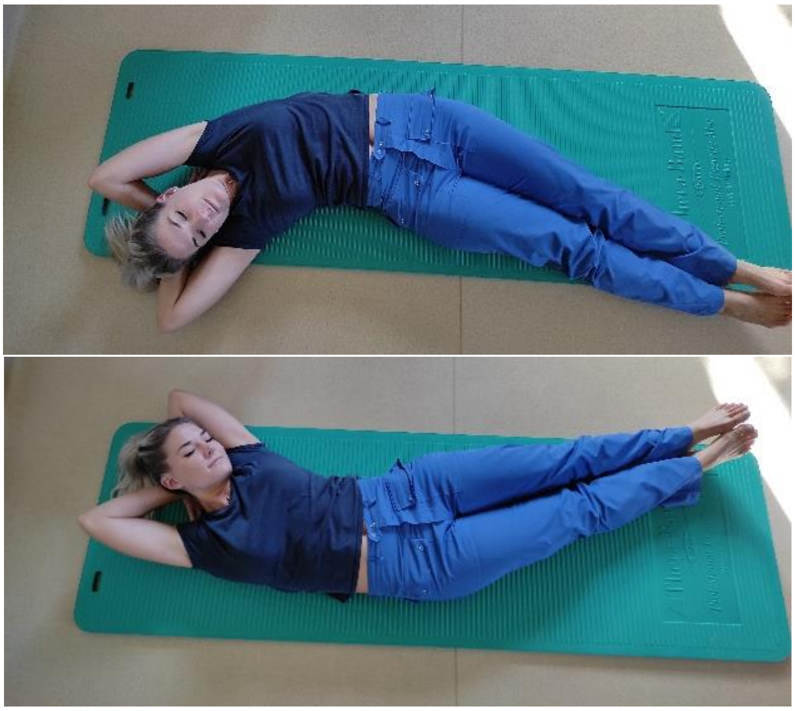

Figure S18. Kingyo Undō ("Goldfish workout" - 金魚運動): Swinging the hips horizontally like fish swimming for about 1 minute.

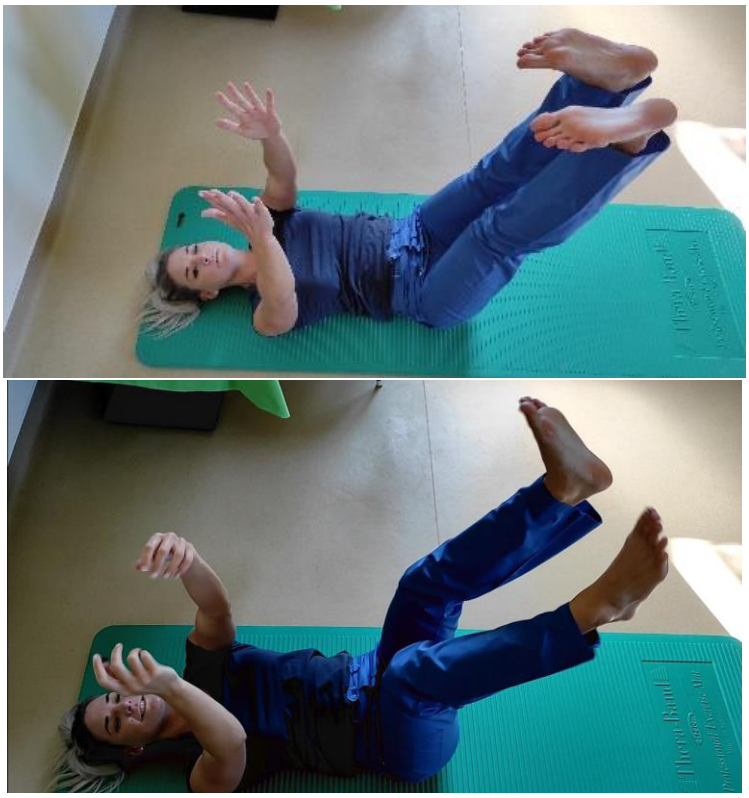

Figure S19. Mōkan undō ("Capillary workout" - 毛管運動): Lying on the back and rising the arms and legs. Vibrating and shaking movements of the limbs for 1 minute.

BATES-SCHNEIDER EXERCISES

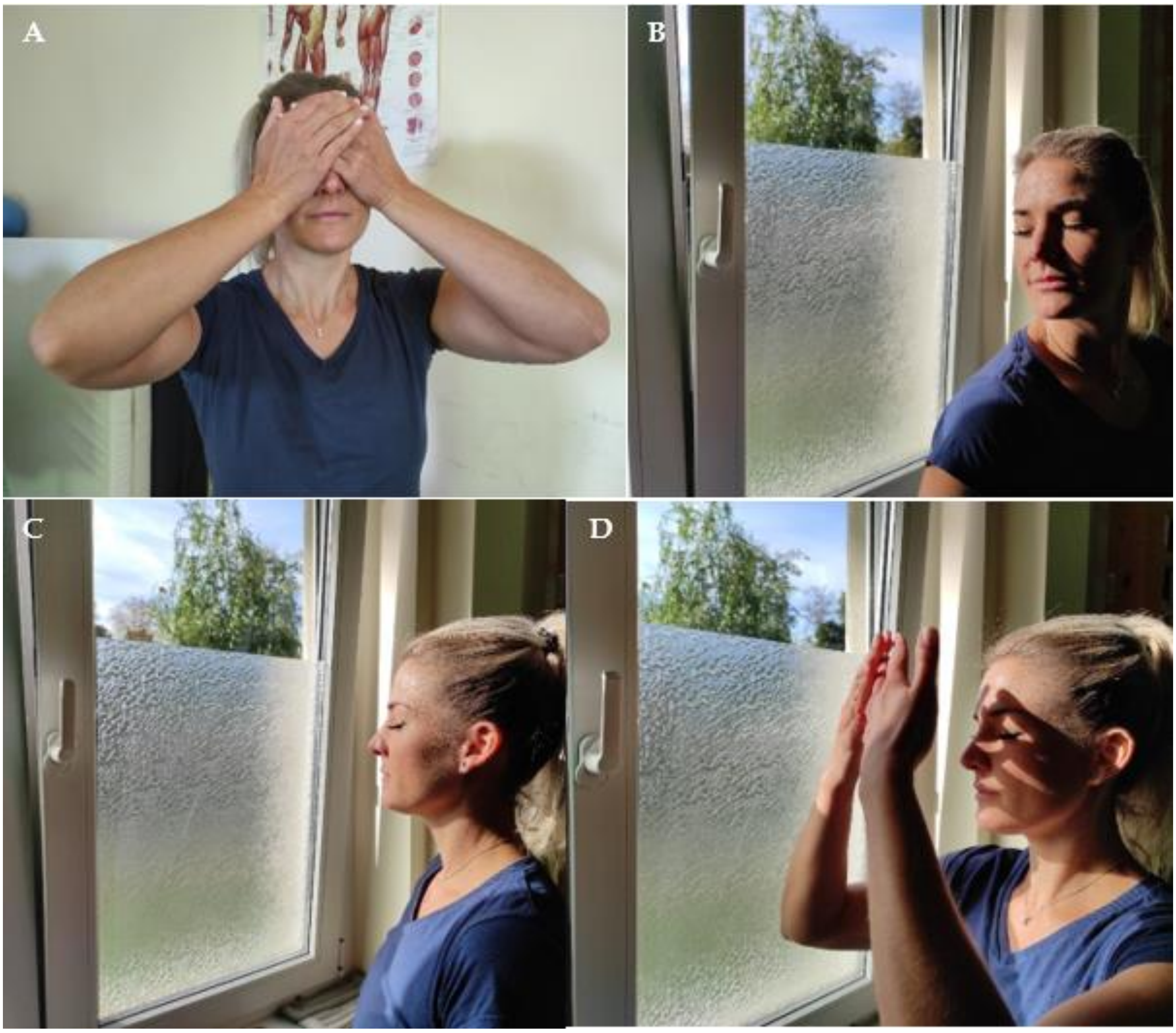

Figure S20. During the palming (**A**) process, both closed eyes are covered with crossed palms in a relaxing position. Natural darkness causes the eyes to relax. In the meantime, visualization and meditation supplemented with breathing exercises can be performed. Sunning firstly starts with the body position back to the sun (**B**) to get used to the sunlight. With the eyes closed, the face is exposed to the sun, turning left and right rhythmically. Later, facing the sunlight, the rhythmic head movement is repeated (**C**), supplemented by the partial shading effect of the palms (**D**), promoting saccadic eye movements.

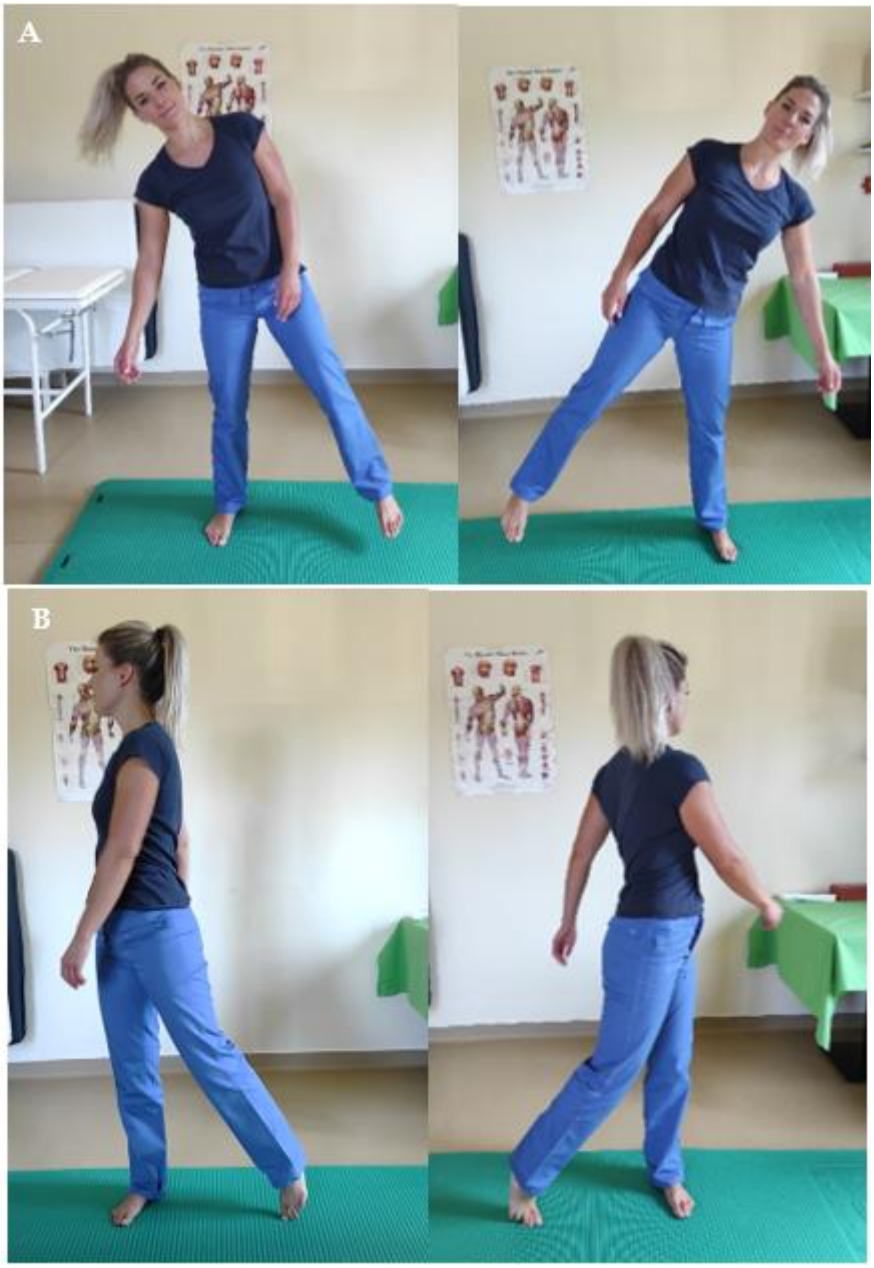

Figure S21. During the rhythmic swaying (**A**) and swinging (**B**) of the whole body, the eyes are forced to focus on a specific target object align stationary on the fovea. Visual information from the environment is gathered through these quick eye movements, which consist of a series of saccades and fixations.
